# Supplementary material for: Organelle landscape analysis using a multiparametric particle-based method
Source: PLoS Biol. 2024 Sep 17;22(9):e3002777. doi: 10.1371/journal.pbio.3002777 (PMC11407678; doi:10.1371/journal.pbio.3002777)
Supplement: S1 Fig — (A) The emission spectra of the fluorophores (Alexa Fluor 405, mTagBFP2, EGFP, Venus, TMR-Star, and Alexa Fluor 594/647/680) used in confocal microscopy. (B) Schematic representation of the equipment employed for 8-color confocal microscopy. Specimens were subjected to excitation by 4 lasers reflected with a dichroic mirror. Fluorescence images were then captured by 4 sets of spectrometers equipped with diffraction gratings and detectors. (C) Montage of fluorescence images obtained by spectral imaging of fluorescently labeled organelle particles. Images acquired by shifting the median wavelength (10-nm width) by 5 nm are aligned from the upper left (411 nm) to the lower right (731 nm). Images acquired by spectrometers 1, 2, 3, and 4 are outlined in purple, cyan, green, and red, respectively. Numbers indicate the mean wavelength of each window. Scale bar, 100 μm. (PDF) [file pbio.3002777.s001.pdf]

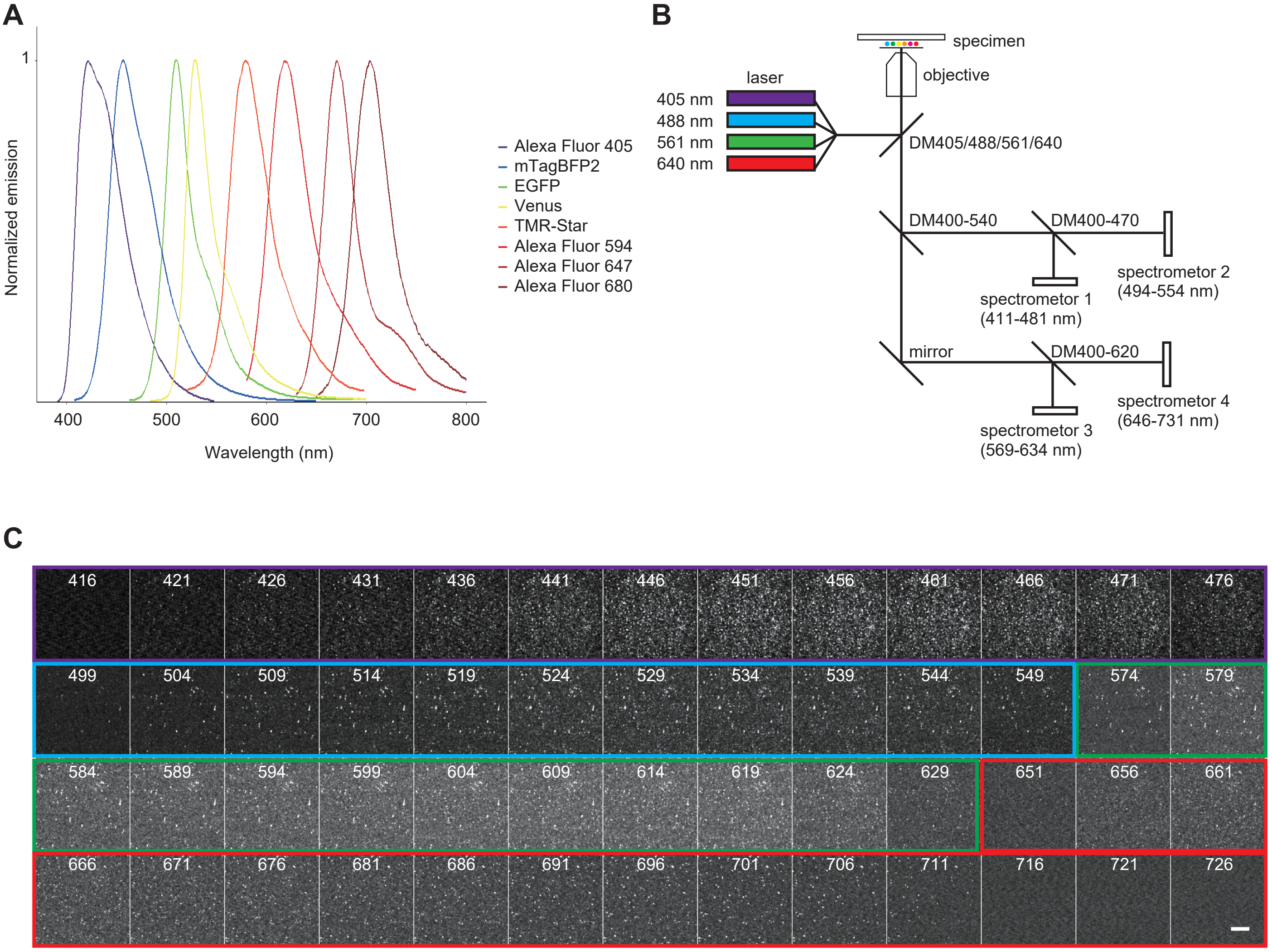

S1 Fig. Spectral imaging of fluorescently labeled organelle particles; related to Fig 1.

(A) The emission spectra of the fluorophores (Alexa Fluor 405, mTagBFP2, EGFP, Venus, TMR-Star, and Alexa Fluor 594/647/680) used in confocal microscopy. (B) Schematic representation of the equipment employed for eight-color confocal microscopy. Specimens were subjected to excitation by four lasers reflected with a dichroic mirror. Fluorescence images were then captured by four sets of spectrometers equipped with diffraction gratings and detectors. (C) Montage of fluorescence images obtained by spectral imaging of fluorescently labeled organelle particles. Images acquired by shifting the median wavelength (10-nm width) by 5 nm are aligned from the upper left (411 nm) to the lower right (731 nm). Images acquired by spectrometers 1, 2, 3, and 4 are outlined in purple, cyan, green, and red, respectively. Numbers indicate the mean wavelength of each window. Scale bar, 100 μm.
