# Supplementary material for: Organelle landscape analysis using a multiparametric particle-based method
Source: PLoS Biol. 2024 Sep 17;22(9):e3002777. doi: 10.1371/journal.pbio.3002777 (PMC11407678; doi:10.1371/journal.pbio.3002777)
Supplement: S2 Fig — (A) PCA plots of the data obtained from 8-color fluorescent images of organelle particles derived from HeLa cells. Particles were colored according to the fluorescence intensity of each marker. The maximum fluorescence intensity of each marker was set to 100%. (B) The data from 3 independent experiments were plotted on UMAP spaces. The numbers of particles plotted in each experiment were as follows: Experiment 1, 10,749; Experiment 2, 14,286; and Experiment 3, 11,160. Data obtained from 8-color fluorescent images of particles of 7 typical organelles derived from HeLa cells can be found in S1 Data. (PDF) [file pbio.3002777.s002.pdf]

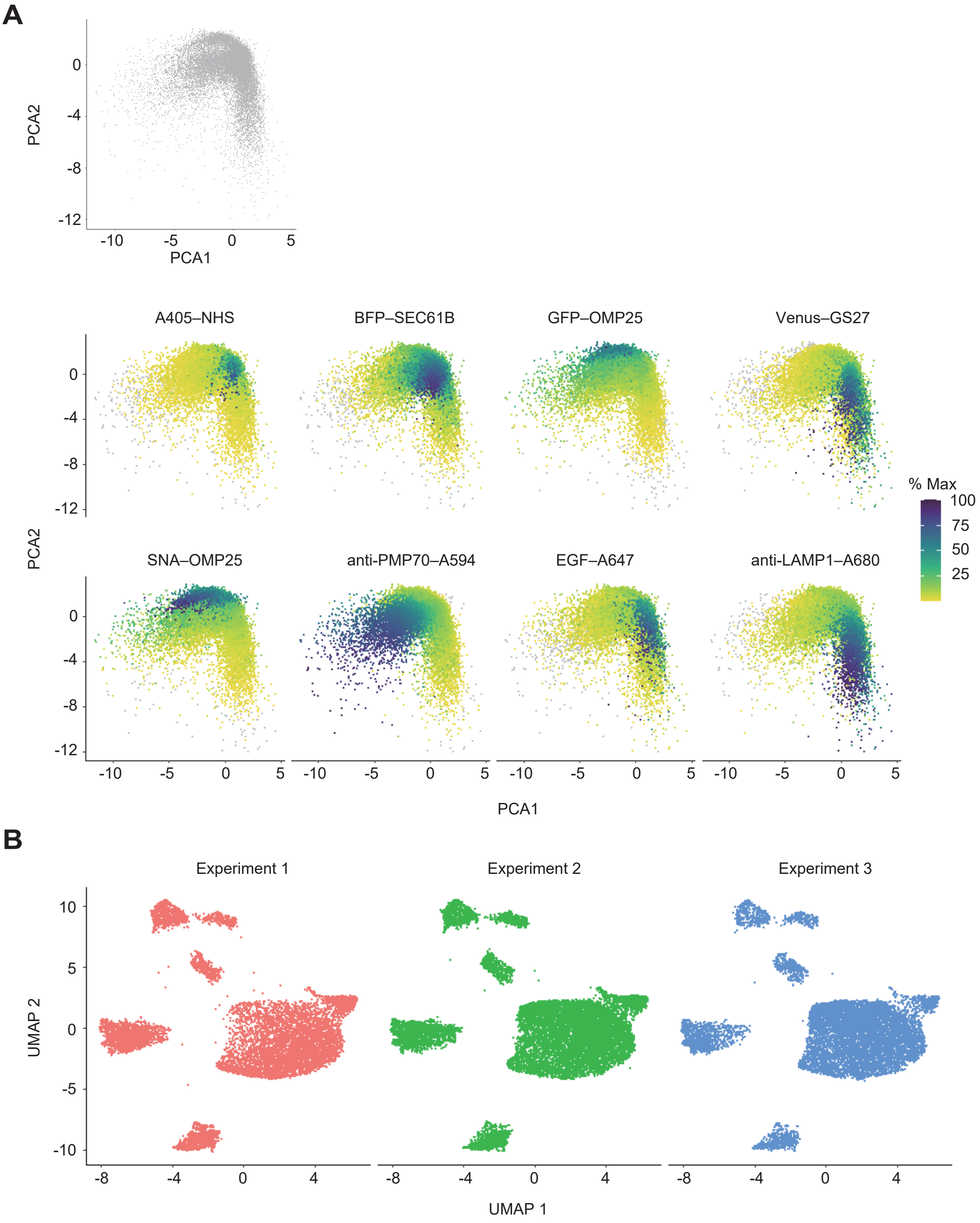


**S2 Fig. Dimension reduction of the eight-color data; related to Fig 2.**

(A) Principal component analysis (PCA) plots of the data obtained from eight-color fluorescent images of organelle particles derived from HeLa cells. Particles were colored according to the fluorescence intensity of each marker. The maximum fluorescence intensity of each marker was set to 100%. (B) The data from three independent experiments were plotted on uniform manifold approximation and projection (UMAP) spaces. The numbers of particles plotted in each experiment were as follows: Experiment 1, 10,749; Experiment 2, 14,286; and Experiment 3, 11,160. Data obtained from eight-color fluorescent images of particles of seven typical organelles derived from HeLa cells can be found in S1 Data.
