# Supplementary material for: Organelle landscape analysis using a multiparametric particle-based method
Source: PLoS Biol. 2024 Sep 17;22(9):e3002777. doi: 10.1371/journal.pbio.3002777 (PMC11407678; doi:10.1371/journal.pbio.3002777)
Supplement: S3 Fig — (A, B) Montage of fluorescence images obtained by spectral imaging of fluorescently labeled organelle particles. Organelle particles from HeLa cells expressing BFP–SEC61B and GFP–VAMP7 (A) or PEX3–GFP (B) were labeled with Alexa405–NHS and both anti-PMP70–A594 and anti-LAMP1–A680 antibodies and are shown as in S1C Fig. Scale bar, 100 μm. (C, D) Unmixing results of the fluorescent spectral images in A and B, respectively. Scale bar, 50 μm. (PDF) [file pbio.3002777.s003.pdf]

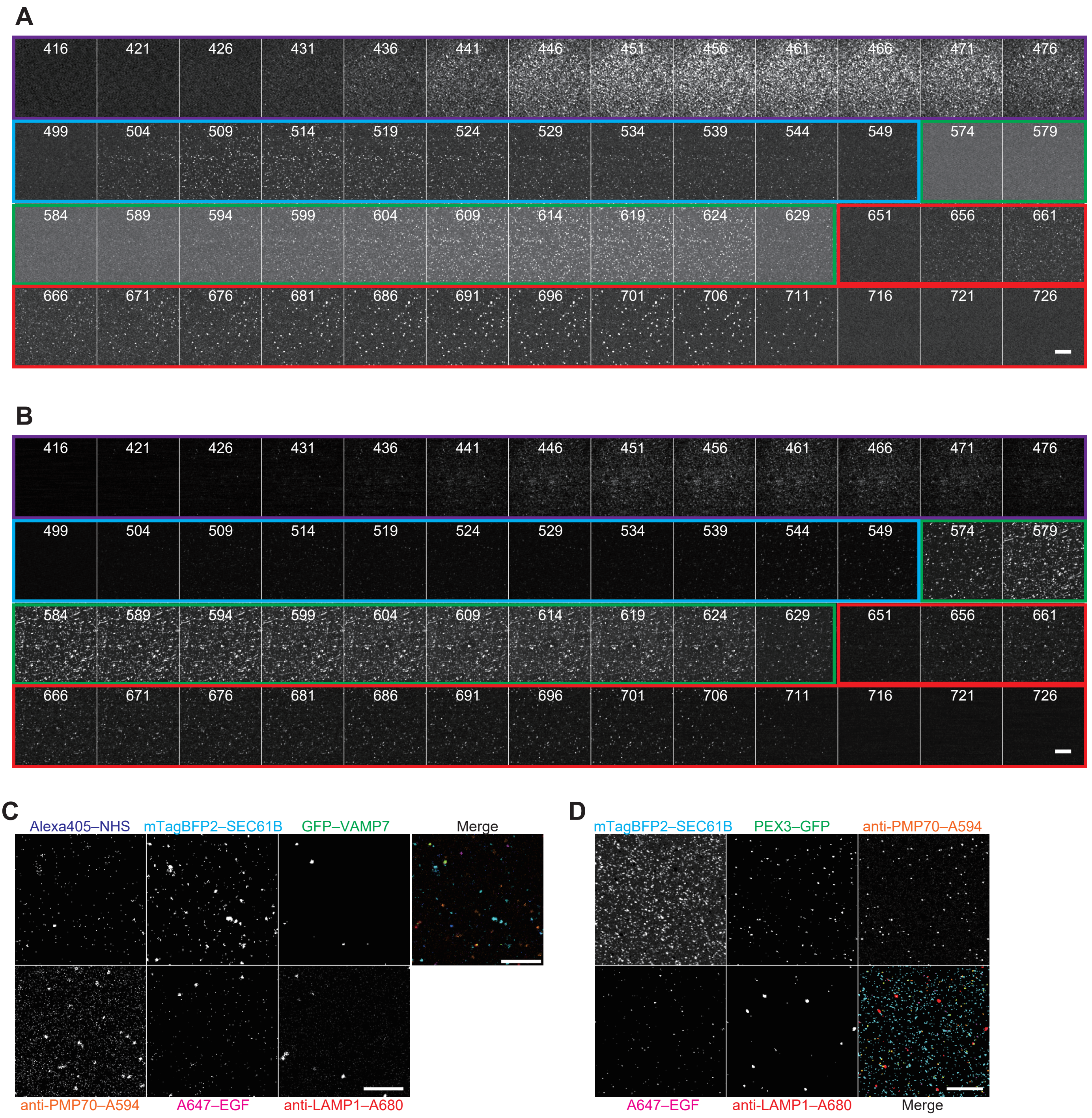

S3 Fig. Spectral imaging and linear unmixing of the images of organelle particles labeled with GFP–VAMP7 or GFP–PEX3.

(A, B) Montage of fluorescence images obtained by spectral imaging of fluorescently labeled organelle particles. Organelle particles from HeLa cells expressing BFP–SEC61B and GFP–VAMP7 (A) or PEX3–GFP (B) were labeled with Alexa405–NHS and both anti-PMP70–A594 and anti-LAMP1–A680 antibodies and are shown as in S1C Fig. Scale bar, 100 μm. (C, D) Unmixing results of the fluorescent spectral images in A and B, respectively. Scale bar, 50 μm.
