# Supplementary material for: Organelle landscape analysis using a multiparametric particle-based method
Source: PLoS Biol. 2024 Sep 17;22(9):e3002777. doi: 10.1371/journal.pbio.3002777 (PMC11407678; doi:10.1371/journal.pbio.3002777)
Supplement: S4 Fig — (A, B) UMAP embedding of the data obtained from 6-color fluorescent images of particles of 5 typical organelles derived from HeLa cells described in S3 Fig (expressing GFP–VAMP7 (A) or PEX3–GFP (B)). The numbers of particles classified in each cluster in A were as follows: Cluster 1, 3,711; Cluster 2, 3,063; Cluster 3, 2,724; Cluster 4, 1,044; Cluster 5, 786; Cluster 6, 317. The numbers of particles classified in each cluster in B were as follows: Cluster 1, 33,167; Cluster 2, 3,553; Cluster 3, 1,926. (C, D) The intensities of the fluorescent markers. Particles were colored according to the fluorescence intensity of each marker. The maximum fluorescence intensity in each marker was set to 100%. Data obtained from 6-color fluorescent images of particles of 5 typical organelles derived from HeLa cells stably expressing GFP–VAMP7 and PEX3–GFP can be found in S3 and S4 Data, respectively. (PDF) [file pbio.3002777.s004.pdf]

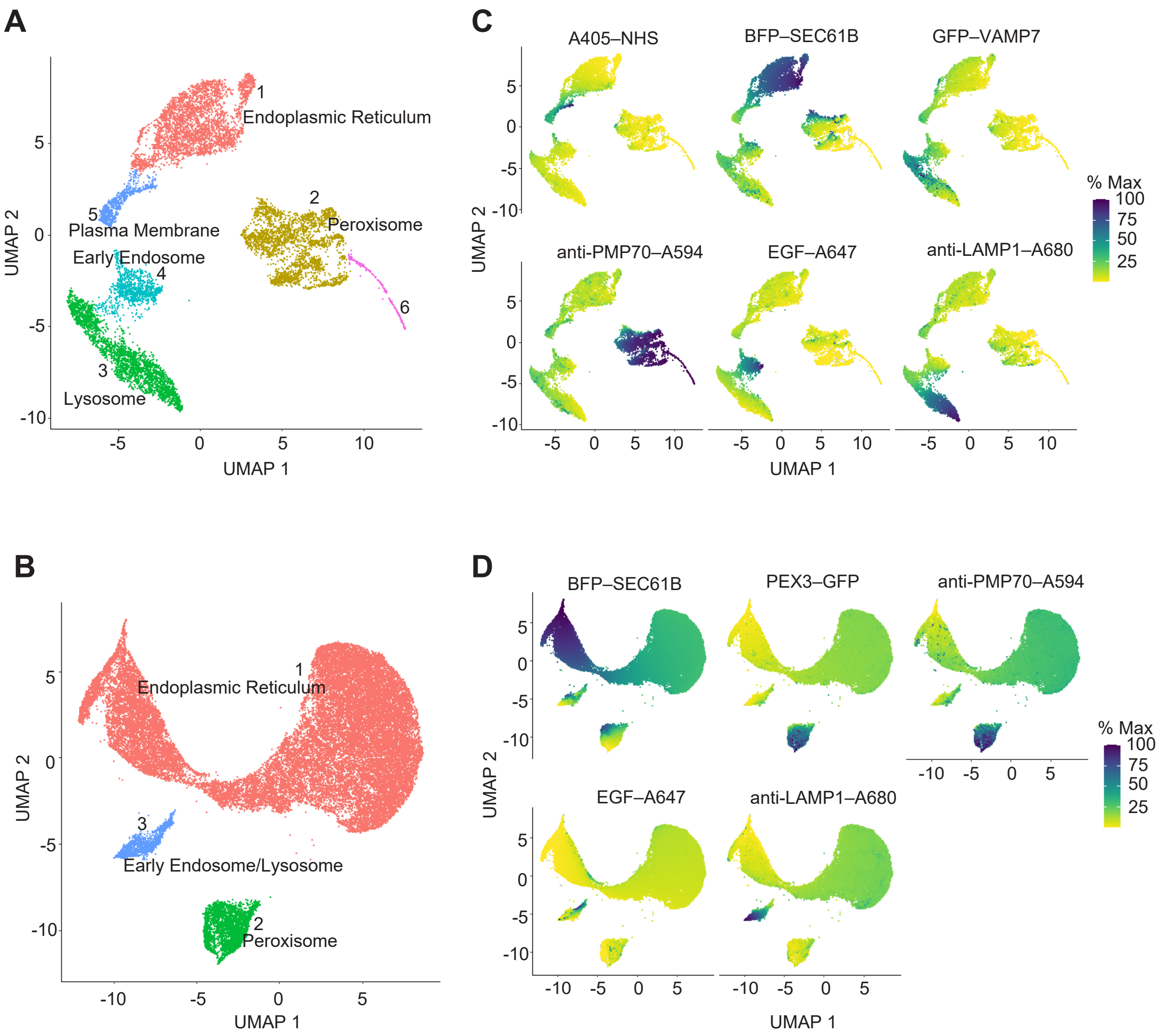


**S4 Fig. Multi-parametric single-particle analysis of typical organelles containing GFP–VAMP7 or PEX3–GFP.**

(A, B) Uniform manifold approximation and projection (UMAP) embedding of the data obtained from six-color fluorescent images of particles of five typical organelles derived from HeLa cells described in S3 Fig (expressing GFP–VAMP7 (A) or PEX3–GFP (B)). The numbers of particles classified in each cluster in A were as follows: Cluster 1, 3,711; Cluster 2, 3,063; Cluster 3, 2,724; Cluster 4, 1,044; Cluster 5, 786; Cluster 6, 317. The numbers of particles classified in each cluster in B were as follows: Cluster 1, 33,167; Cluster 2, 3,553; Cluster 3, 1,926. (C, D) The intensities of the fluorescent markers. Particles were colored according to the fluorescence intensity of each marker. The maximum fluorescence intensity in each marker was set to 100%. Data obtained from six-color fluorescent images of particles of five typical organelles derived from HeLa cells stably expressing GFP–VAMP7 and PEX3–GFP can be found in S3 Data and S4 Data, respectively.
