# Supplementary material for: Organelle landscape analysis using a multiparametric particle-based method
Source: PLoS Biol. 2024 Sep 17;22(9):e3002777. doi: 10.1371/journal.pbio.3002777 (PMC11407678; doi:10.1371/journal.pbio.3002777)
Supplement: S5 Fig — (A) Montage of fluorescence images obtained by spectral imaging of fluorescently labeled organelle particles. HEK293T cells expressing mTagBFP2–SEC61B and GFP–OMP25 were loaded with A647–EGF, and their organelle particles were labeled with anti-PMP70–A594 and anti-LAMP1–A680 antibodies, as shown in S1C Fig. Scale bar, 100 μm. (B) Unmixing results of the fluorescence spectral images in A. Scale bar, 50 μm. (PDF) [file pbio.3002777.s005.pdf]

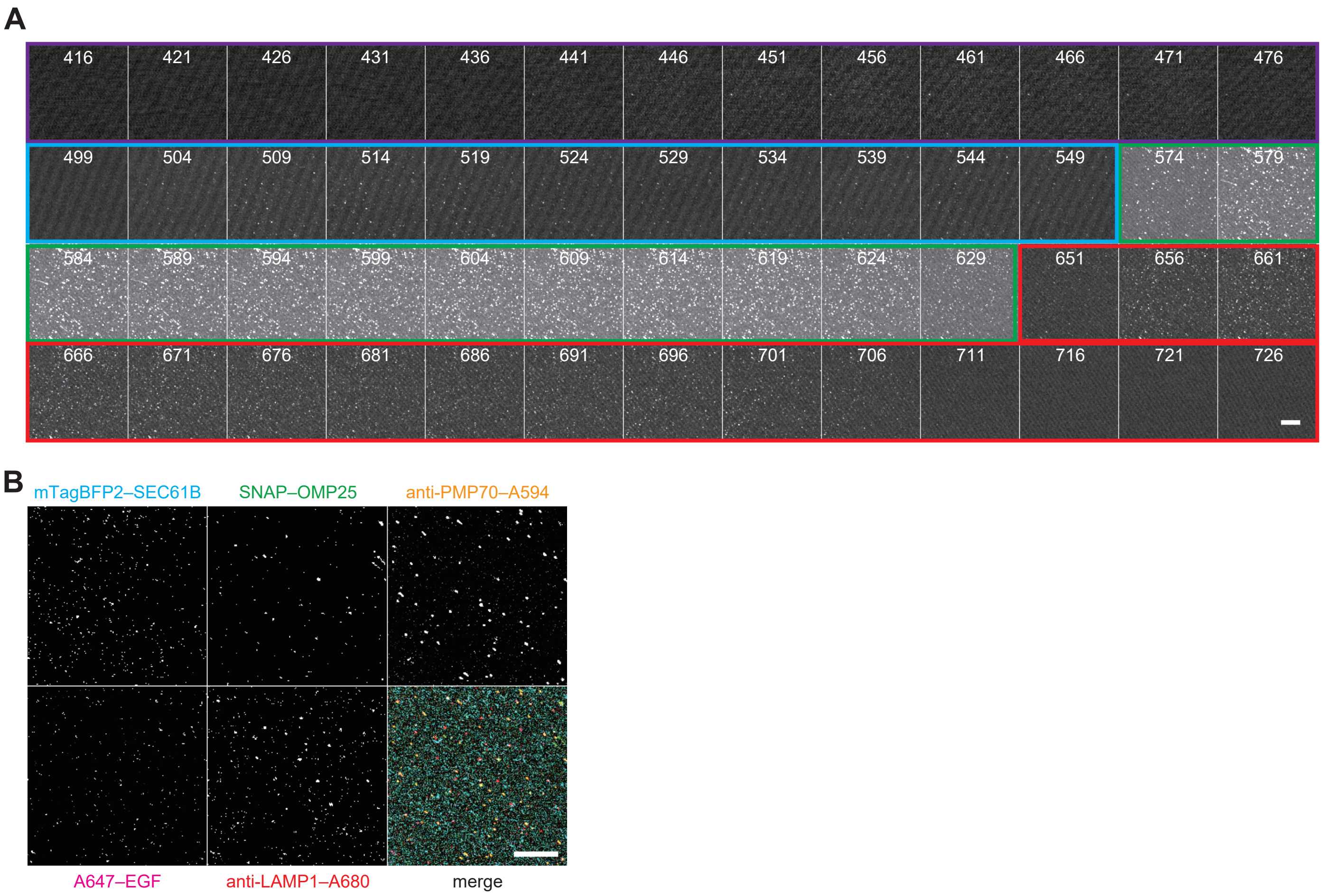

S5 Fig. Spectral imaging and linear unmixing of the images of organelle particles from HEK293T cells.

(A) Montage of fluorescence images obtained by spectral imaging of fluorescently labeled organelle particles. HEK293T cells expressing mTagBFP2–SEC61B and GFP–OMP25 were loaded with A647–EGF, and their organelle particles were labeled with anti-PMP70–A594 and anti-LAMP1–A680 antibodies, as shown in S1C Fig. Scale bar, 100 μm. (B) Unmixing results of the fluorescence spectral images in A. Scale bar, 50 μm
