# Supplementary material for: Organelle landscape analysis using a multiparametric particle-based method
Source: PLoS Biol. 2024 Sep 17;22(9):e3002777. doi: 10.1371/journal.pbio.3002777 (PMC11407678; doi:10.1371/journal.pbio.3002777)
Supplement: S6 Fig — (A) UMAP embedding of the data obtained from 6-color fluorescent images of particles of 5 typical organelles derived from HEK293T cells. The numbers of particles classified in each cluster were as follows: Cluster 1, 3,690; Cluster 2, 1,450; Cluster 3, 1,416; Cluster 4, 642; Cluster 5, 577. (B) The intensities of the fluorescent markers. Particles are colored according to the fluorescence intensity of each marker. The maximum fluorescence intensity in each marker was set to 100%. Data obtained from 5-color fluorescent images of particles of 4 typical organelles derived from HEK293T cells can be found in S5 Data. (PDF) [file pbio.3002777.s006.pdf]

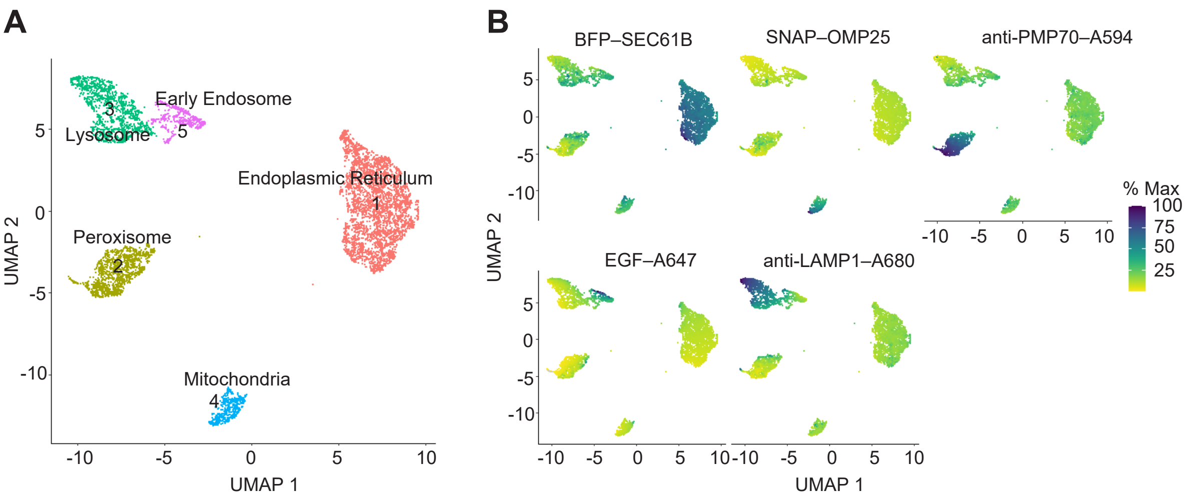

S6 Fig. Multi-parametric single-particle analysis of typical organelle particles from HEK293T cells.

(A) Uniform manifold approximation and projection (UMAP) embedding of the data obtained from six-color fluorescent images of particles of five typical organelles derived from HEK293T cells. The numbers of particles classified in each cluster were as follows: Cluster 1, 3,690; Cluster 2, 1,450; Cluster 3, 1,416; Cluster 4, 642; Cluster 5, 577. (B) The intensities of the fluorescent markers. Particles are colored according to the fluorescence intensity of each marker. The maximum fluorescence intensity in each marker was set to 100%. Data obtained from five-color fluorescent images of particles of four typical organelles derived from HEK293T cells can be found in S5 Data.
