# Supplementary material for: Organelle landscape analysis using a multiparametric particle-based method
Source: PLoS Biol. 2024 Sep 17;22(9):e3002777. doi: 10.1371/journal.pbio.3002777 (PMC11407678; doi:10.1371/journal.pbio.3002777)
Supplement: S7 Fig — (A) Fluorescent images of HeLa cells expressing BFP–SEC61B, GFP–ER–mito [ER–mitochondria contact site marker, ERj1(1–200)–V5–GFP1–10 and TOMM70(1–70)–3×FLAG–GFP11], and SNAP–OMP25. Scale bars, 10 μm and 2 μm (inset). (B) Montage of fluorescence images obtained by spectral imaging of fluorescently labeled organelle particles. Images were acquired and are shown as in Extended Data Fig 1C. (C) Unmixing results of fluorescent spectral images in B. Scale bar, 100 μm. (PDF) [file pbio.3002777.s007.pdf]

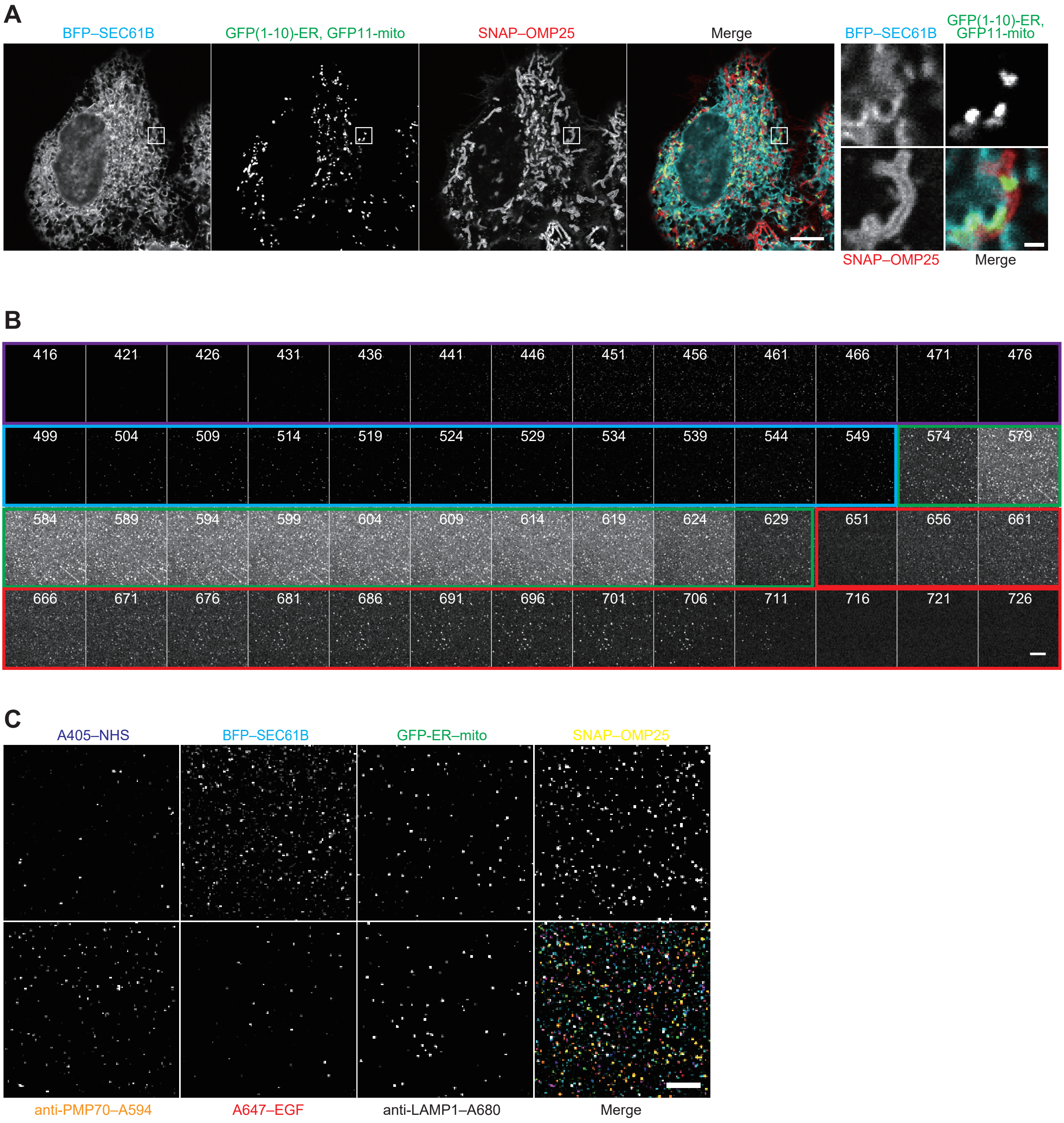

S7 Fig. Spectral imaging and linear unmixing of images of organelle particles containing an ER–mitochondria contact site marker; related to Fig 3. (A) Fluorescent images of HeLa cells expressing BFP–SEC61B, GFP-ER–mito [ER–mitochondria contact site marker, ERj1(1–200)–V5–GFP1–10 and TOMM70 (1–70)–3xFLAG–GFP11], and SNAP–OMP25. Scale bars, 10 μm and 2 μm (inset). (B) Montage of fluorescence images obtained by spectral imaging of fluorescently labeled organelle particles. Images were acquired and are shown as in S1C Fig. (C) Unmixing results of fluorescent spectral images in B. Scale bar, 100 μm.
