# Supplementary material for: Organelle landscape analysis using a multiparametric particle-based method
Source: PLoS Biol. 2024 Sep 17;22(9):e3002777. doi: 10.1371/journal.pbio.3002777 (PMC11407678; doi:10.1371/journal.pbio.3002777)
Supplement: S8 Fig — (A) Intensities of fluorescent markers from the reference data shown in Fig 3. Particles were colored according to the fluorescence intensity of each marker. The maximum fluorescence intensity in each marker was set to 100%. (B) UMAP embedding of the data of the reference obtained from 3 independent experiments. The numbers of particles plotted on each experiment were as follows: Experiment 1, 10,387; Experiment 2, 13,830; and Experiment 3, 10,641. Data obtained from 8-color fluorescent images of particles labeled with 6 organelle markers as references can be found in S6 Data. (PDF) [file pbio.3002777.s008.pdf]

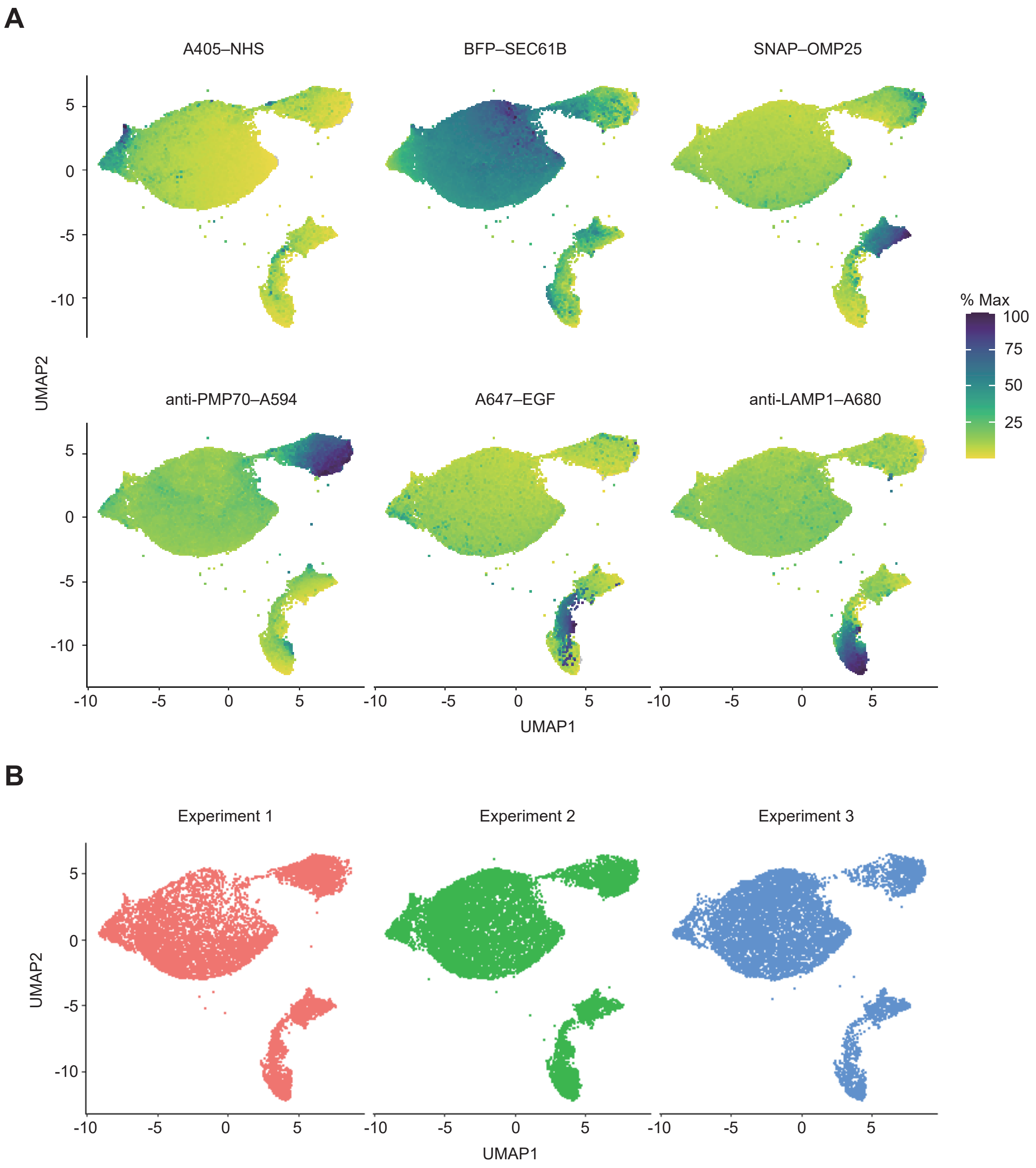

S8 Fig. Distribution of organelle markers of the reference data in uniform manifold approximation and projection (UMAP) space; related to Fig 3. (A) Intensities of fluorescent markers from the reference data shown in Fig 3. Particles were colored according to the fluorescence intensity of each marker. The maximum fluorescence intensity in each marker was set to 100%. (B) UMAP embedding of the data of the reference obtained from three independent experiments. The numbers of particles plotted on each experiment were as follows: Experiment 1, 10,387; Experiment 2, 13,830; and Experiment 3, 10,641. Data obtained from eight-color fluorescent images of particles labeled with six organelle markers as references can be found in S6 Data.
