# Supplementary material for: Organelle landscape analysis using a multiparametric particle-based method
Source: PLoS Biol. 2024 Sep 17;22(9):e3002777. doi: 10.1371/journal.pbio.3002777 (PMC11407678; doi:10.1371/journal.pbio.3002777)
Supplement: S9 Fig — (A) Intensities of the fluorescent markers from the query data. Red arrows indicate split-GFP-positive particles. (B) UMAP embedding of the data of the query obtained from 3 independent experiments. The numbers of particles plotted in each experiment were as follows: Experiment 1, 7,245; Experiment 2, 4,160; and Experiment 3, 6,065. Data of the experiments with the ER–mitochondrial contact site marker as query can be found in S7 Data. (PDF) [file pbio.3002777.s009.pdf]

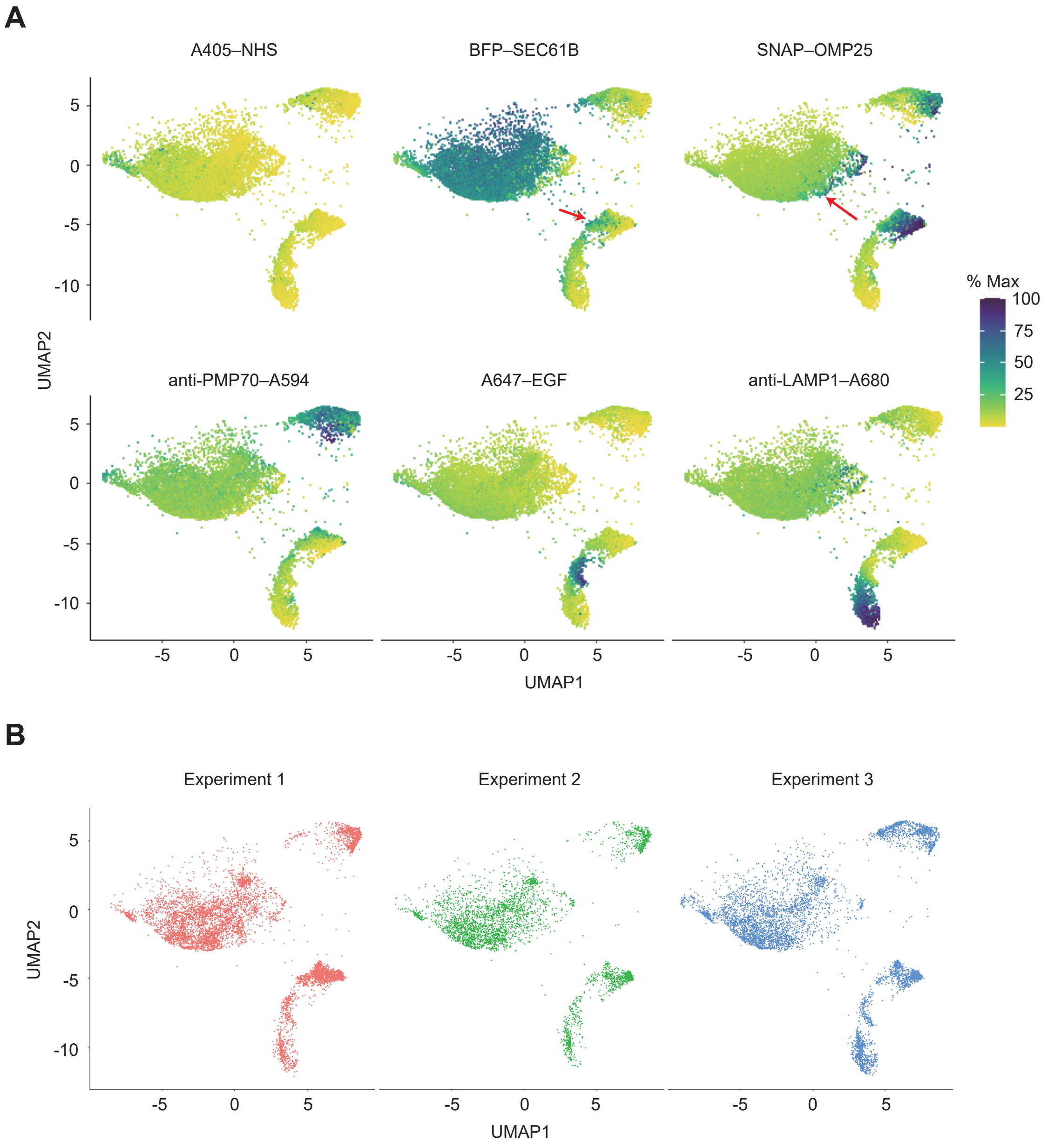

S9 Fig. Distribution of organelle markers of the query data of Fig. 3 in uniform manifold approximation and projection (UMAP) space; related to Fig 3.

(A) Intensities of the fluorescent markers from the query data. Red arrows indicate split-GFP-positive particles. (B) UMAP embedding of the data of the query obtained from three independent experiments. The numbers of particles plotted in each experiment were as follows: Experiment 1, 72,45; Experiment 2, 4,160; and Experiment 3, 6,065. Data of the experiments with the ER–mitochondrial contact site marker as query can be found in S7 Data.
