# Supplementary material for: Organelle landscape analysis using a multiparametric particle-based method
Source: PLoS Biol. 2024 Sep 17;22(9):e3002777. doi: 10.1371/journal.pbio.3002777 (PMC11407678; doi:10.1371/journal.pbio.3002777)
Supplement: S10 Fig — (A, B) Montage of fluorescence images obtained by spectral imaging of fluorescently labeled endocytic particles without (A) and with (B) wortmannin treatment. Images were acquired and are shown as in S1C Fig. (C, D) Unmixing results of the fluorescent spectral images in A and B. (E) Histogram of the signal intensity of particles from cells treated or untreated with A647–EGF and A594–transferrin. The 99th percentile point for untreated samples is indicated by the red dashed line. (PDF) [file pbio.3002777.s010.pdf]

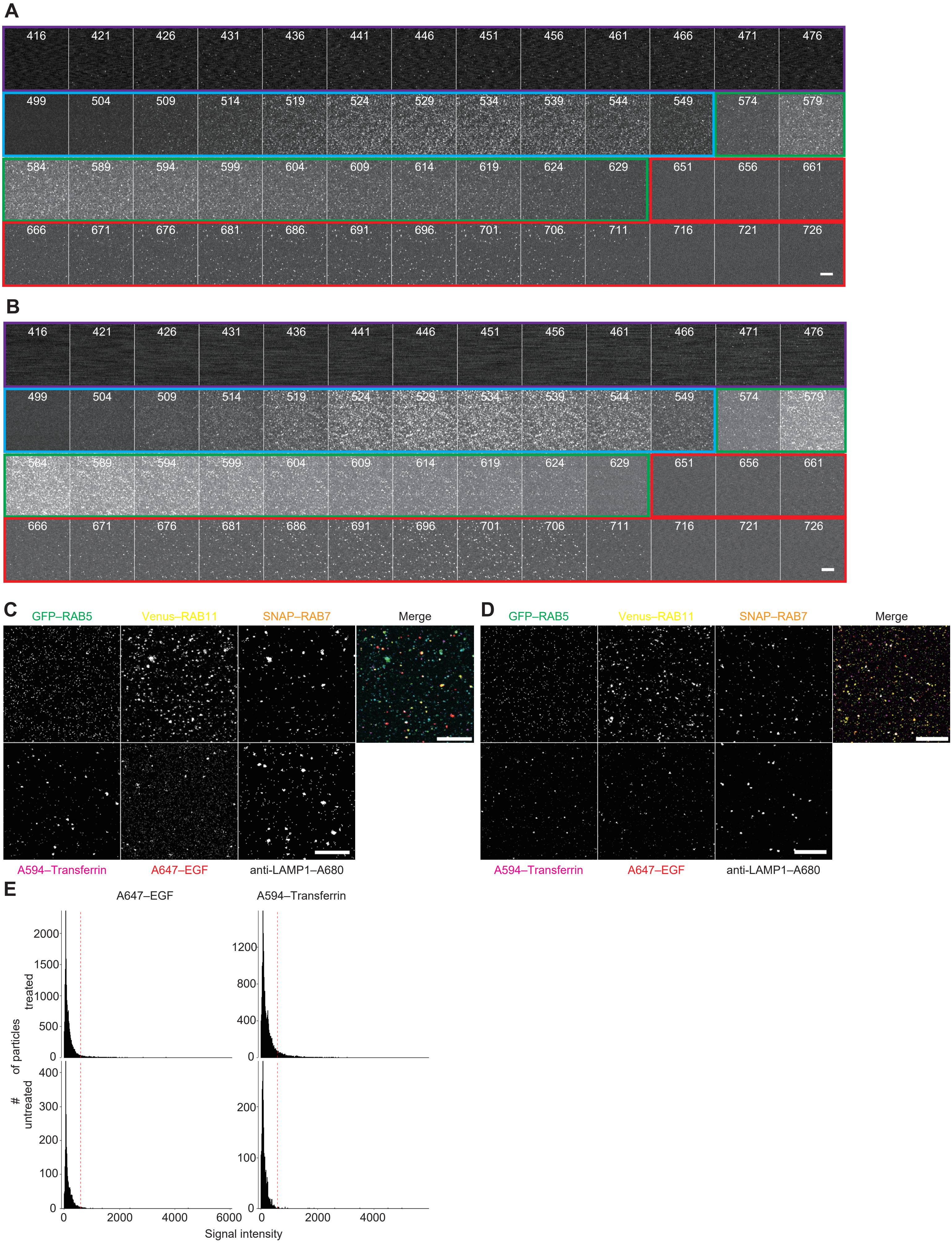

S10 Fig. Spectral imaging and linear unmixing of the images of organelle particles labeled with endocytosis-related markers; related to Fig 4. (A, B) Montage of fluorescence images obtained by spectral imaging of fluorescently labeled endocytic particles without (**A**) and with (**B**) wortmannin treatment. Images were acquired and are shown as in S1C Fig. (C. D) Unmixing results of the fluorescent spectral images in **A** and **B**. (E) Histogram of the signal intensity of particles from cells treated or untreated with A647–EGF and A594–Transferrin. The 99th percentile point for untreated samples is indicated by the red dashed line.
