# Supplementary material for: Organelle landscape analysis using a multiparametric particle-based method
Source: PLoS Biol. 2024 Sep 17;22(9):e3002777. doi: 10.1371/journal.pbio.3002777 (PMC11407678; doi:10.1371/journal.pbio.3002777)
Supplement: S11 Fig — (A) Particles from each replicate separately plotted on the UMAP data shown in Fig 4C. The numbers of particles plotted on each experiment were as follows: Experiment 1, 2,893; Experiment 2, 5,791; and Experiment 3, 8,429. (B) Distribution of each replicate in Clusters 1–7. Data obtained from fluorescent images of particles of endosomes containing EGF or transferrin can be found in S8 Data. (PDF) [file pbio.3002777.s011.pdf]

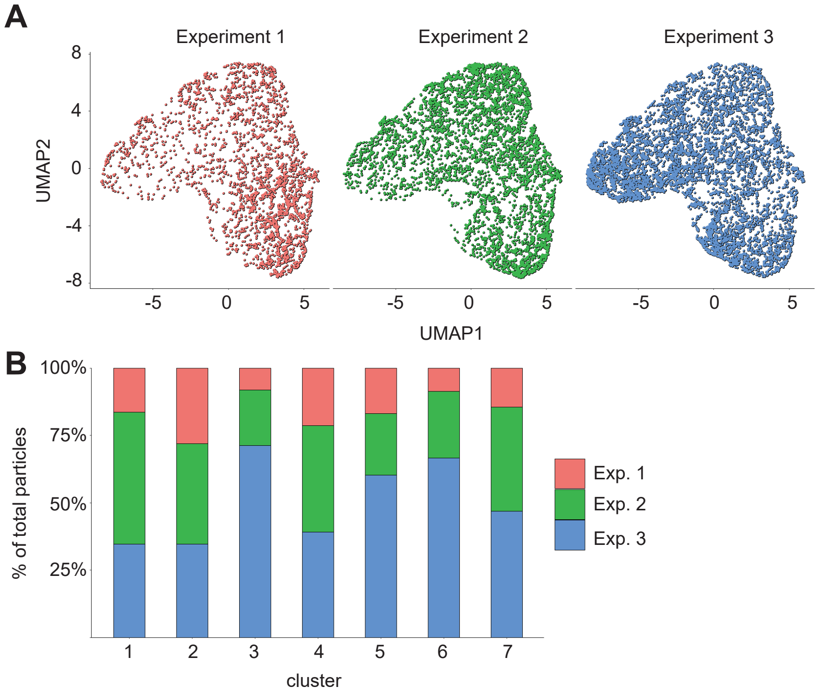

S11 Fig. Reproducibility of the data and marker intensities in each cluster in the endocytosis analysis; related to Fig 4.

(A) Particles from each replicate separately plotted on the uniform manifold approximation and projection (UMAP) data shown in Fig 4C. The numbers of particles plotted on each experiment were as follows: Experiment 1, 2,893; Experiment 2, 5,791; and Experiment 3, 8,429. (B) Distribution of each replicate in Clusters 1–7. Data obtained from fluorescent images of particles of endosomes containing EGF or transferrin can be found in S8 Data.
