# Supplementary material for: Organelle landscape analysis using a multiparametric particle-based method
Source: PLoS Biol. 2024 Sep 17;22(9):e3002777. doi: 10.1371/journal.pbio.3002777 (PMC11407678; doi:10.1371/journal.pbio.3002777)
Supplement: Attachment — Submitted filename: 20240123_revision-plan_upload.pdf [file pbio.3002777.s022.pdf]

Revision Plan

Review COMMONS

Manuscript number: RC-2023-02193
Corresponding author(s): Ikuko Koyama-Honda, Noboru Mizushima

1. General Statements [optional]

2. Description of the planned revisions

Reviewer #1, Comment (1)
To further demonstrate the unique power of this new method, the authors should test cells under known stimulation altering the dynamics of organelles. For instance, wortmannin can blocks the conversion from early endosomes to late endosomes. By doing that, the potential of this new method will be endorsed.
Response:
We thank Reviewer #1 for the positive comments. We will add an experiment using wortmannin to block the process of endocytosis at a specific stage, as part of the experiments analyzing the process of endocytosis.

Reviewer #2, Comment (1)
Initially, I think the current set of cell lines and labels should be extended also to include a wider set. The current limited set raises the question if the method authors report is also applicable to other cell lines, or if it only feasible with overexpressed markers. Including different cell lines with different labels would make the study more convincing and comprehensive.
Response:
We thank Reviewer #2 for this constructive comment. Regarding cell types, we will conduct experiments with HEK293T cells in addition to HeLa cells, labeling at least five different types of typical organelles. In our method, as shown in Figure 1a and 5a, we have already used not only overexpressed markers but also fluorescently labeled ligands (EGF-Alexa, transferrin-Alexa) and antibodies against endogenous proteins (anti-PMP70, anti-LAMP1), as well as direct labeling of cell membrane proteins (Alexa-NHS). Therefore, there are no significant limitations with respect to organelle labeling methods.

Reviewer #2, Comment (3)
Most organelle markers the isolation of organelles are based on are overexpressed in the cells: endoplasmic reticulum (ER, mTagBFP2 (BFP)-SEC61B), mitochondria (GFP-OMP25 and SNAP-OMP25), and the Golgi (Venus-GS27). This raises significant

questions about the native state relevance of the reported results, and how well they represent the endogenous processes.

Response:

We will add experiments analyzing the behavior of both endogenous and exogenous markers for the same organelles, for example, anti-LAMP1 antibody and VAMP7-GFP for lysosomes, and anti-PMP70 antibody and PEX16-GFP for peroxisomes.

3. Description of the revisions that have already been incorporated in the transferred manuscript

Please insert a point-by-point reply describing the revisions that were already carried out and included in the transferred manuscript. If no revisions have been carried out yet, please leave this section empty.

The following revisions were accomplished and indicated in red in the transferred manuscript.

Reviewer #1, Comment (2)

The authors should include more details about how to avoid signal crosstalk between adjacent fluorescent channels.

Response:

In the Methods section, we have added the following sentences to Lines 398-405.

"In order to avoid signal crosstalk between adjacent fluorescence channels, eight fluorophores with distinct spectral distances were selected, and the samples were irradiated sequentially with lasers in the order from the longest wavelength, i.e., fluorescence from 646 to 731 nm was excited by a 640 nm laser, fluorescence from 569 to 634 nm was excited by a 561 nm laser, fluorescence from 494 to 554 nm was excited by a 488 nm laser, and fluorescence from 411 to 481 nm was excited by a 405 nm laser, as shown in Extended Data Fig. 1b."

Reviewer #2, Comment (2)

It is surprising that the authors explicitly list already the limitations of fluorescence microscopy and super-resolution microscopy in the second paragraph of their introduction, however present a method fully dependent on fluorescence labelling and imaging methods. Actually their approach takes away the spatial information of FM approaches, and further makes the approach prone to the limitations they state.
They are also not fully fair about the limitation they state for Electron microscopy, as newly developed approaches (e.g. doi:10.1093/micmic/ozad067.1091; doi:10.1126/science.aay3134) widely extend the limited field of view and sampling

capacity of EM. I recommend the authors to state the potential advantage/superiority of the reported method rather than stating the unclear limitations of the existing powerful methods.

Response:

Regarding fluorescence microscopy, it appears that our description was inadequate and misled the reviewers. There is no problem with fluorescence microscopy itself. What we intended to convey was that “when attempting to detect individual organelles ‘in cells’, there are limitations in the resolution of fluorescence microscopy because organelles are densely packed”. To clarify this point, we have added the words “in cells” to the text on Line 49. Also, we thank Reviewer #2 for informing us about the high-speed 3D electron microscopy. We have cited the indicated papers in the text at Lines 54-55 and mention that “except for the recently developed high-throughput electron microscopy”.

Reviewer #2, Comment (5)

The significance of biochemical and cellular processes being spatially regulated cellular organelles, and the roles of specific organelles in diseases from cancer to neurodegeneration are continuously being discovered and appreciated. Therefore development of methods reporting on the structure and function of organelles is important to accelerate these studies. In the reported method, however, the ultrastructure (as in Fib 1b) and the spatial information of the cellular organelles are inherently lost. The method falls in between a biochemical and a microscopic approach, however the advantages are not clearly portrayed. I recommend the authors to carefully and explicitly state where their method would be the method of choice rather than a biochemistry, mass spectroscopy, or microscopy approach. The authors should critically consider such an experiment as a proof-of-concept case.

Response:

We thank Reviewer #2 for the valuable suggestion. We added the following to the Discussion (Lines 267-277).

“A further potential application of our method would be to measure how the levels of key molecules in an organelle change during its differentiation or maturation. For example, the levels of PI4P and syntaxin 17 change during autophagosome maturation (Shinoda et al. eLife Preprint Review doi.org/10.7554/eLife.92189.1), which can be better demonstrated by this method using multiple markers for each stage of autophagosome formation and maturation, PI4P, and syntaxin17 because autophagosomes at different stages coexist in cells. In such cases, our single-particle analysis method, which examines the state of individual autophagosomes, would be more appropriate than biochemical methods that examine averages. In addition, it is difficult to quantitatively analyze many organelle structures in cells using fluorescence microscopy. Our particle-based analysis method can overcome this problem.”

Revision Plan

Reviewer #3, Comment (1)
I would like to start with a positive suggestion. The authors completely miss out on the opportunity to promote their approach by not relying on any type of fixation. In most multiplexing experiments, the first major challenge is to find antibodies that work well for imaging. The second challenge is then to find antibodies that work well under the same fixation conditions. The authors present a multiplexing approach that is completely independent of fixation. I suggest discussing this in the manuscript and promoting the approach in that regard.
Response:
We thank Reviewer #3 for pointing out the advantages of our method. We have added "Our method that is independent of fixation is advantageous for the optimization of the staining condition." to the Discussion (Lines 298-299).

Reviewer #3, Comment (2)
I am wondering what defines the 'resolution' of this approach. I assume it is a combination of the sonication time -the longer the cell is sonicated, the smaller the fragments are - and the density of particles on the coverslip. What are the limits here? How does this affect the UMAP analysis? I would encourage the authors to discuss this in the manuscript.
Response:
The particle density on a coverslip can be easily reduced by simply diluting the particles in a buffer solution. Therefore, there is no density limit, which is an advantage of a cell-free system. To improve the resolution within a single organelle, for example, to separate distinct subdomains, as the reviewer mentioned, we can prolong the sonication time to make the particles smaller. However, since this will reduce the signal-to-background ratio and destroy organelle contacts, we used the sonication conditions as mild as possible. To investigate organelle subdomains and fragile contacts, the sonication conditions need to be optimized carefully, but we think that these will be future work.

We have added the above discussion to the Discussion (Lines 288-293).
"Also, to improve the resolution within a single organelle, for example, to separate distinct subdomains, we can prolong the sonication time to make the particles smaller. However, since this will reduce the signal-to-background ratio and may destroy organelle contacts, we used the sonication conditions as mild as possible. To investigate organelle subdomains and fragile contacts, the sonication conditions need to be optimized carefully."

We do not think that prolonged sonication will affect the UMAP analysis because relative fluorescent signals of each particle would not change. However, as mentioned above, too strong sonication would worsen the signal-to-noise ratio, resulting in poor clustering.

Reviewer #3, Comment (3)
The only real control the authors present are the correlative light and electron microscopy (CLEM) three images in Figure 1b, which seems very minimalistic for a very central and essential control experiment. How many of these control images did the authors take? Is there possibly a second method for a control experiment to link the fluorescence readout to an organelle fragment (e.g., purification or pulldown)?
Response:
Since all the markers we used are well-established, we believe that there is no concern about the fluorescence readouts to the organelle fragments. We have cited the following papers in Lines 84-85.

SEC61B: Rapoport, T. A., Jungnickel, B. & Kutay, U. Protein transport across the eukaryotic endoplasmic reticulum and bacterial inner membranes. Annu Rev Biochem 65, 271–303 (1996).

OMP25: Horie, C., Suzuki, H., Sakaguchi, M. & Mihara, K. Characterization of signal that directs C-tail-anchored proteins to mammalian mitochondrial outer membrane. Mol Biol Cell 13, 1615–1625 (2002).

GS27: Hay, J. C. et al. Localization, Dynamics, and Protein Interactions Reveal Distinct Roles for ER and Golgi SNAREs. J Cell Biol 141, 1489–1502 (1998).

Fusella, A., Micaroni, M., Di Giandomenico, D., Mironov, A. A. & Beznoussenko, G. V. Segregation of the Qb-SNAREs GS27 and GS28 into Golgi Vesicles Regulates Intra-Golgi Transport. Traffic 14, 568–584 (2013).

Although it is relatively easy to identify mitochondria-derived particles by EM based on their size and the presence of cristae-like structures (indeed we see many examples), it is more challenging for other organelles (because they appear simple vesicles). This is why we showed only mitochondria in Fig. 1b. Furthermore, the main purpose of this EM image is to show membrane contacts between the ER and mitochondria (related to Fig. 3).

Reviewer #3, Comment (4)

Line 37-41: Could the authors please strengthen these statements with an appropriate citation (e.g., a review)?
Response:
We have cited the textbook Molecular Biology of THE CELL (the 6th edition, Chapter 12 and Chapter 13) in Lines 37 and 41.

Reviewer #3, Comment (5)
Line 51: The statement, “Super-resolution microscopy could partially solve the resolution problem, but it is currently limited to four-color imaging,” is incorrect. Agasti et al. demonstrated up to nine target multiplexed super-resolved imaging with DNA-PAINT in 2017 (https://pubs.rsc.org/en/content/articlehtml/2017/sc/c6sc05420j). Additionally, two papers currently on Biorxiv demonstrate 12 target and 30 target multiplexed super-resolution imaging with FLASH-PAINT (https://www.biorxiv.org/content/10.1101/2023.05.17.541061v1.abstract) and SUM-PAINT(https://www.biorxiv.org/content/10.1101/2023.05.17.541210v1.abstract). Please cite these papers accordingly.
Response:
We thank Reviewer #3 for notifying us of these important studies. We have rewritten the sentence on Lines 51-52 to read “Although multicolor imaging has been attempted with super-resolution microscopy (references of the indicated papers), it only partially solves the issue of resolution.”

Reviewer #3, Comment (6)
The authors use spectral unmixing to overcome the limit of spectral multiplexing. While this has been demonstrated to work well for less than ten targets, it does not scale to multiplexing experiments with more than ten target species. I suggest that the authors discuss in the discussion part of the manuscript the potential of DNA-based multiplexed imaging, such as CODEX or DNA-PAINT, in combination with the presented approach.
Response:
In the Discussion (Lines 295-298), we have added the sentence “Current fluorescent particle detection uses spectral multiplexing, but this method has only been able to detect up to eight colors. Methods such as CODEX or DNA-PAINT with wide-field type illumination could significantly increase the number of targets”.

Reviewer #3, Comment (7)
Regarding the spectral unmixing approach, please cite previous work described in the literature (e.g.,https://www.nature.com/articles/nature22369, or earlier work).
Response:
We thank Reviewer #3 for informing us. We have cited it in Line 72.

Reviewer #3, Comment (8)
By using spectral unmixing for multiplexing, this method is limited to confocal due to spectral detection needs and therefore limited in throughput. It would be beneficial if it could work with wide-field type illumination. This could substantially increase the throughput, which is another reason why I think it would be important to discuss sequential multiplexing.
Response:
We agree with the Reviewer's comment. We have added the discussion to Lines 295-298 as described in our response to Reviewer #3, Comment (6).

Reviewer #3, Comment (10)
Line 27 & 253: Please drop the term 'intuitive' or explain better what you mean by intuitive. For me, UMAP is certainly a very useful tool, but it is not at all what I would describe as intuitive.
Response:
We have deleted 'intuitive' in all seven places and rewritten them (Lines 27, 43, 58, 72, 180, 231, and 253).

Reviewer #3, Comment (11)
Lastly, I want to mention that the authors state they used ChatGPT, DeepL, and DeepL Write for translation from Japanese to English. I appreciate their honesty.
Response:
We thank Reviewer #3 for the comment.

4. Description of analyses that authors prefer not to carry out

Reviewer #2, Comment (4)
For the application on endosomes, can the authors state what is the new information enabled by their method? They study the very trafficking of EGF and Transferrin, 2 widely used endosomal cargoes with very well characterized trafficking steps, and show they are trafficked through Rab5/7 and Rab11 positive endosomes, respectively. This recapitulates the existing information, however falls short in delivering new insight. The authors can use these cargoes for proof-of-concept, but I would recommend to extend their study with less exploited cargoes to represent the potential of the reported method to deliver new information.

Response:

We thank Reviewer #2 for the positive suggestion about the potential of our method to provide new information. However, to demonstrate new biological insights, it would take a lot of time and delay the provision of our methodology, so we would like to submit this manuscript as a Methods paper with the proof-of-concept data. As the Author Guidelines for the PLOS Biology's article type 'Methods' state that new biological insights are not a requirement for consideration, we believe that our manuscript should meet these criteria.

Reviewer #3, Comment (9)

To image contact sites, the authors use split GFP. There have been discussions that split GFP might, in some cases, facilitate the process that is supposed to be measured, in this case, the formation of contact sites. I suggest using at transient version of split GFP, called split fast, for follow-up experiments in the authors' next papers (https://www.nature.com/articles/s41467-019-10855-0).

Response:

We thank Reviewer #3 for providing this information. We will do it as suggested in the next paper.
