# Supplementary material for: Organelle landscape analysis using a multiparametric particle-based method
Source: PLoS Biol. 2024 Sep 17;22(9):e3002777. doi: 10.1371/journal.pbio.3002777 (PMC11407678; doi:10.1371/journal.pbio.3002777)
Supplement: Attachment — Submitted filename: Rebuttal_letter_20240507.pdf [file pbio.3002777.s024.pdf]

Response to Reviewers:

Reviewer #1 (Evidence, reproducibility and clarity (Required)):

In this manuscript, the authors report a novel and simple method to analyze the heterogeneity of various organelles. After imaging a large set of fluorescent-marker-labeled organelles, cluster analysis is adapted for illuminating the dynamics of organelles. Through this novel method, the authors are able to report organelle contact, which previously can only be observed by super-resolution imaging. This is method could significantly accelerate future discoveries at the cellular level. The manuscript is well written and has the potential be published in high-ranking journals, after a minor revision.
To further demonstrate the unique power of this new method, the authors should test cells under known stimulation altering the dynamics of organelles. For instance, wortmannin can blocks the conversion from early endosomes to late endosomes. By doing that, the potential of this new method will be endorsed.

Response:
We thank Reviewer #1 for the positive comments and valuable suggestion. We have added an experiment using wortmannin to block the process of endocytosis, as part of the experiments analyzing the process of endocytosis. Wortmannin partially inhibited EGF degradation and transferrin recycling, as reported. These results further validate our method.
We have also added the corresponding results (Figs 4 and 5) and the following sentences to the manuscript.

Line 234: “To examine the impact of perturbations on EGF endocytosis, we treated cells with wortmannin, an inhibitor of the class III PI3 kinase complex, for 15 min before EGF addition. In wortmannin-treated cells, a decrease in the bright EGF population was not observed, indicating a delay in degradation (Fig 4B, right upper panel).”

Line 254: “Next, we embedded the data from the wortmannin-treated cells into the UMAP space by metric learning, using the data from untreated cells as a reference (Fig 4F). The data were separated into seven clusters, resembling the

clustering of the untreated cells. However, the number of particles in Clusters 2, 3, 6, and 7 was reduced, suggesting that the inhibition of the recycling and degradation pathways by wortmannin was adequately represented in the UMAP space.”

Line 280: “Upon treatment with wortmannin, EGF was predominantly distributed in Clusters 1 and 5, while EGF in Clusters 6 and 7 was reduced, indicating inhibition of transport to late endosomes and lysosomes (Fig 5C). Transferrin was also predominantly distributed in Clusters 1 and 5, with a decrease in Clusters 2 and 3 (Fig 5D). This is consistent with previous research showing that transferrin localizes to RAB5-positive structures instead of RAB11-positive structures upon PI3 kinase inhibition (Ref #38–41).”

With the addition of the new figures, the previous Fig 4F has been moved to Fig 5A and B.

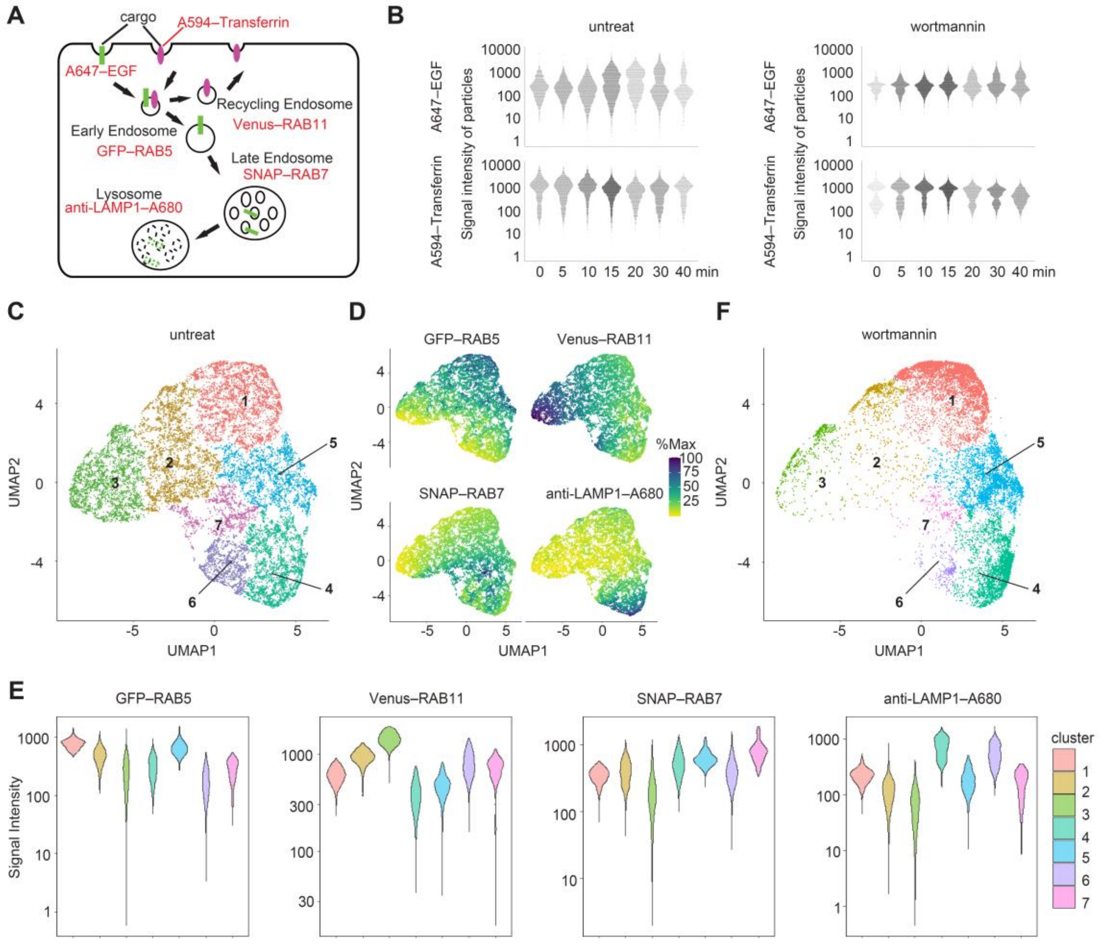

Fig 4. Multi-parametric particle-based analysis of endocytic vesicles containing EGF or transferrin. (A) Schematic illustration of cells used for the analysis of endocytic compartments. Green and magenta indicate A647–EGF and A594–transferrin signals, respectively. Cells were treated with wortmannin or left untreated, followed by treatment with A647–EGF and A594–transferrin for 30 min at 4°C, washing, and incubation at 37°C for 0, 5, 10, 15, 20, 30, and 40 min. (B) Signal intensities of Alexa647–EGF and Alexa594–transferrin on particles positive for either EGF or transferrin over the time course (after the shift to 37°C). For wortmannin-untreated cells, the numbers of particles plotted at each time point were as follows: 0 min, 1,820; 5 min, 2,281; 10 min, 2,758; 15 min, 4,394; 20 min, 1,946; 30 min, 2,681; 40 min, 1,046. For wortmannin-treated cells, the particle numbers were as follows: 0 min, 461; 5 min, 2,050; 10 min, 3,415; 15 min, 3,329; 20 min, 1,534; 30 min, 2,369; 40 min, 2,017. (C) Uniform manifold approximation and projection (UMAP) embedding of 17,113 data points obtained from fluorescent images of particles of endosomes containing EGF or transferrin. The numbers of particles classified in each cluster were as follows: Cluster 1, 3,347; Cluster 2, 3,092; Cluster 3, 2,959; Cluster 4, 2,869; Cluster 5, 2,423; Cluster 6, 1,374; Cluster 7, 1,049. (D) The intensities of the indicated fluorescent markers on the UMAP space. Particles are colored according to the fluorescence intensity of each marker. The maximum fluorescence intensity of each marker was set as 100%. (E) Violin plots of the signal intensities of the organelle markers on particles in each cluster. (F) A plot of the 15,197 data points of the experiments with the wortmannin treatment as a query using metric learning with the UMAP results in C as reference. The numbers of particles classified in each cluster were as follows: Cluster 1, 6,405; Cluster 2, 897; Cluster 3, 623; Cluster 4, 3,637; Cluster 5, 3,145; Cluster 6, 295; Cluster 7, 173.

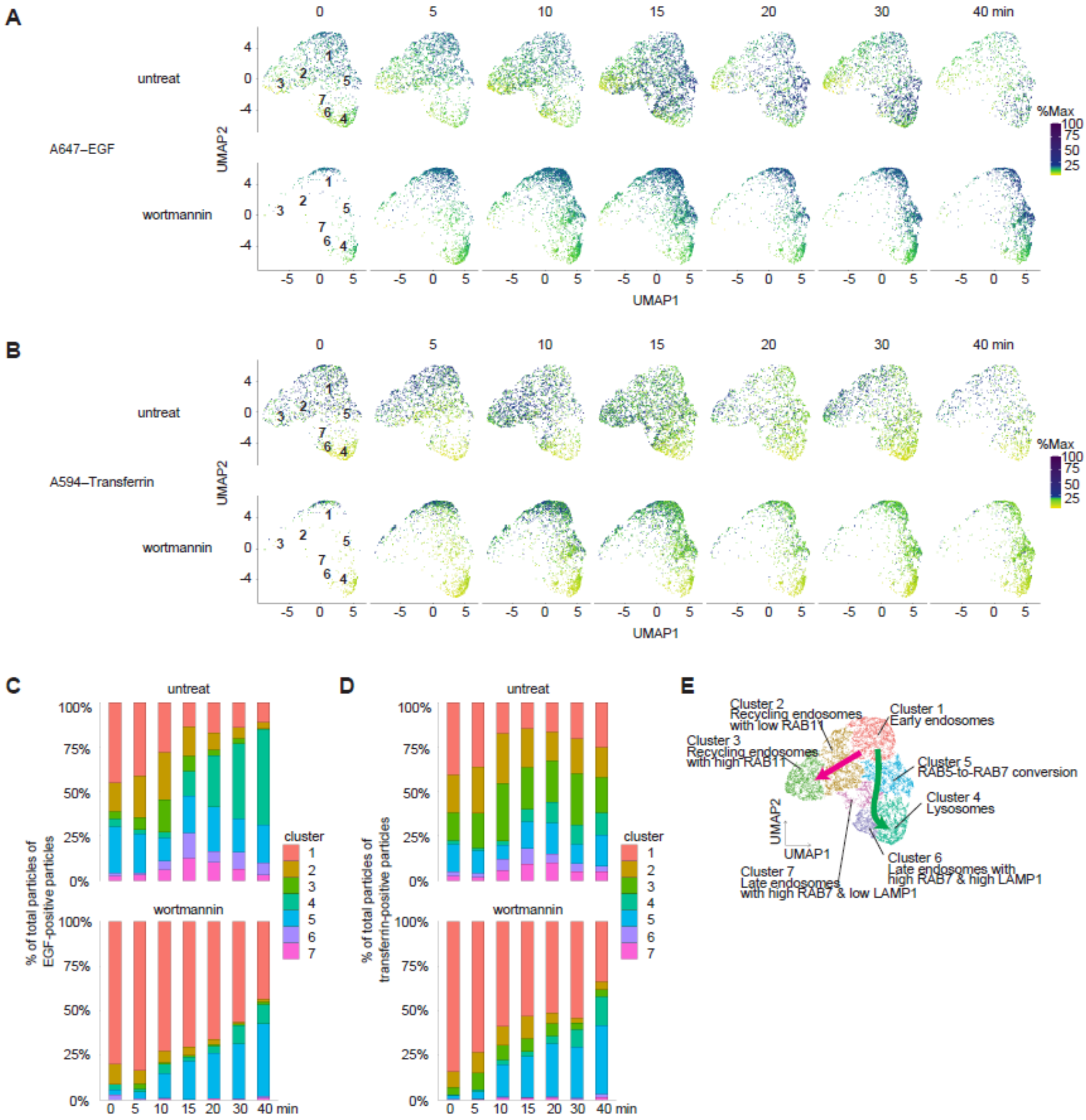

Fig 5. Changes in the distribution of clusters during the endocytic process.

(A, B) Distribution of Alexa647–EGF and A594–transferrin intensities of particles along the time course with or without wortmannin. The colors indicate the signal intensity of EGF or transferrin. The maximum fluorescence intensity of each marker was set to 100%. For visualization, the square root of %max has been plotted. (C, D) Stacked bar graphs showing the proportion of clusters in EGF-positive particles (A) and transferrin-positive particles (B) at each time point (after the shift to 37°C) from wortmannin-treated (lower panels) and untreated (upper panels) cells. (E) Characterization of the clusters and putative pathways depicted in uniform manifold approximation and projection (UMAP) space. The magenta arrow indicates the putative recycling pathway, and the green arrow indicates the degradation pathway.

Minor issue: The authors should include more details about how to avoid signal crosstalk between adjacent fluorescent channels.

Response:
In the Methods section, we have added the following sentences to Lines 445–451.
"In order to avoid signal crosstalk between adjacent fluorescence channels, eight fluorophores with distinct spectral distances were selected, and the samples were irradiated sequentially with lasers in descending order of their wavelengths; that is, fluorescence from 646 to 731 nm was excited by a 640 nm laser, fluorescence from 569 to 634 nm was excited by a 561 nm laser, fluorescence from 494 to 554 nm was excited by a 488 nm laser, and fluorescence from 411 to 481 nm was excited by a 405 nm laser, as shown in S1B Fig."

Reviewer #1 (Significance (Required)):

The comprehensive monitoring of organelle dynamics through the integration of multi-dimensional parameters can proficiently evaluate the condition and prognosticate the destiny of living cells in response to external stimulations. This new multi-dimensional assay reported in this manuscript represents a huge step towards this goal. Since this new method is simple and powerful, cell biologists will quickly start to use this new method for the study of subcellular dynamics.
My lab is also developing a similar approach for organelles based on super-resolution imaging. I would like to congratulate the authors for this beautiful work.

Response:
We thank Reviewer #1 for the positive comment.

Reviewer #2 (Evidence, reproducibility and clarity (Required)):

The manuscript reports a multi-parametric particle-based method for analysis of organelles. The method aims to resolve heterogeneous populations of organelles involved in various cellular processes. They propose to isolate organelles labelled with multiple markers, after homogenization and sonification of the cells, and analyse the resulting particles by fluorescence microscopy using spectral imaging. Afterwards, the authors visualize and analyse the obtained data with dimension reduction techniques.
Even though an interesting approach, the method and presented applications needs major improvisations before it can prove to be impactful for the field
I note some possible improvement points below:

- Initially, I think the current set of cell lines and labels should be extended also to include a wider set. The current limited set raises the question if the method authors report is also applicable to other cell lines, or if it only feasible with overexpressed markers. Including different cell lines with different labels would make the study more convincing and comprehensive.

Response:
We thank Reviewer #2 for this constructive comment. Regarding cell types, we conducted experiments using HEK293T cells in addition to HeLa cells, labeling at least five different types of typical organelles. With our method, as shown in Figs 1A and 5A, we have already used not only overexpressed markers but also fluorescently labeled ligands (EGF–Alexa, transferrin–Alexa) and antibodies against endogenous proteins (anti-PMP70, anti-LAMP1), as well as direct labeling of cell membrane proteins (Alexa–NHS). Therefore, there are no significant limitations with respect to organelle labeling methods.

We have added the results for particles obtained from HEK293T cells to S5 and S6 Fig, and the following sentences to Line 149.
“The applicability of the multi-parameter particle-based analysis was further validated in cell lines other than HeLa cells. HEK293T cells expressing mTagBFP2–SEC61B and GFP–OMP25 were loaded with A647–EGF, and their organelle particles were labeled with anti-PMP70–A594 and anti-LAMP1–A680 antibodies (S5 Fig). The analysis of UMAP embedding revealed clusters based

on the marker proteins (S6 Fig). Thus, organelle particles in HEK293T cells were correctly detected, and their original identities were preserved during the detection process. These findings suggest that the multi-parameter particle-based analysis is applicable across different cell types.”

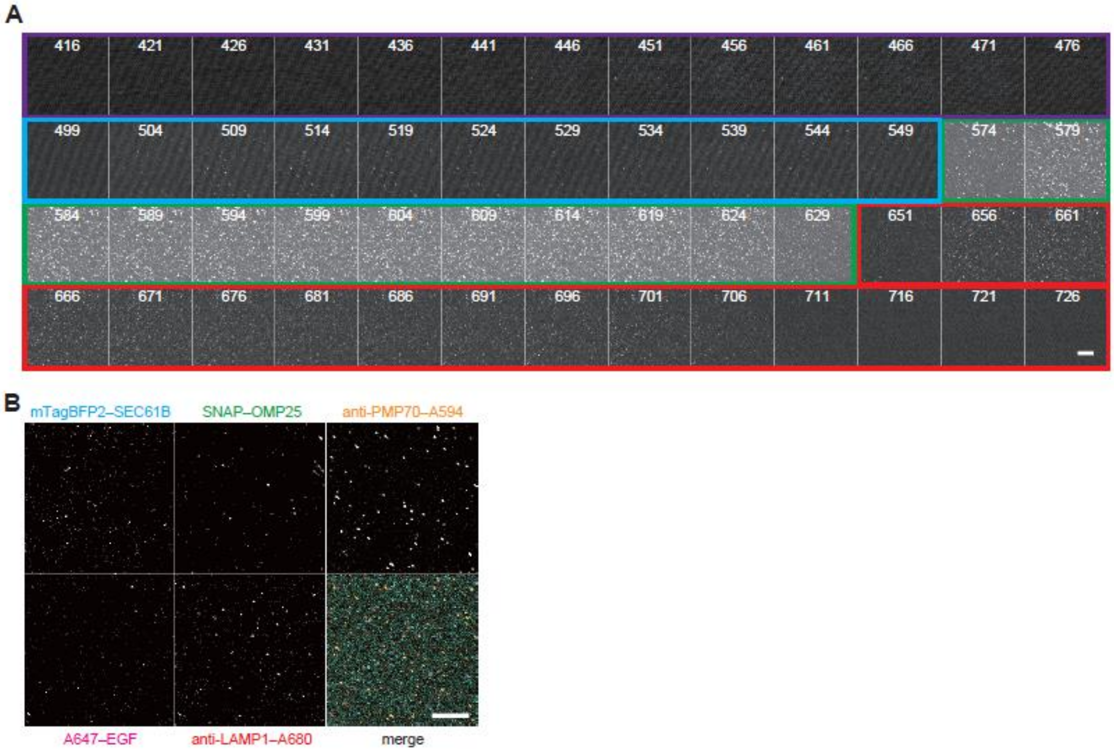

S5 Fig. Spectral imaging and linear unmixing of the images of organelle particles from HEK293T cells.

(A) Montage of fluorescence images obtained by spectral imaging of fluorescently labeled organelle particles. HEK293T cells expressing mTagBFP2–SEC61B and GFP–OMP25 were loaded with A647–EGF, and their organelle particles were labeled with anti-PMP70–A594 and anti-LAMP1–A680 antibodies, as shown in S1C Fig. Scale bar, 100 µm. (B) Unmixing results of the fluorescence spectral images in A. Scale bar, 50 µm.

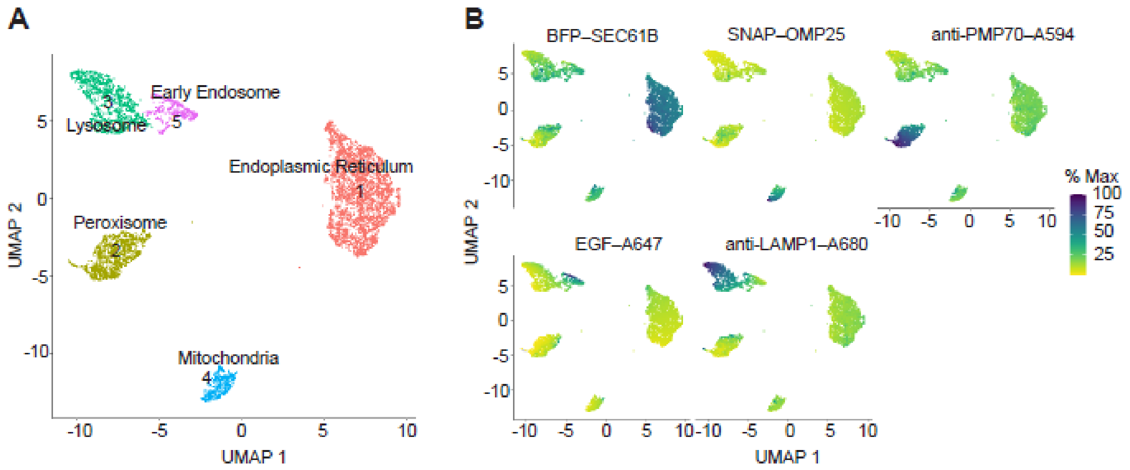

S6 Fig. Multi-parametric single-particle analysis of typical organelle particles from HEK293T cells. (A) Uniform manifold approximation and projection (UMAP) embedding of the data obtained from six-color fluorescent images of particles of five typical organelles derived from HEK293T cells. The numbers of particles classified in each cluster were as follows: Cluster 1, 3,690; Cluster 2, 1,450; Cluster 3, 1,416; Cluster 4, 642; Cluster 5, 577. (B) The intensities of the fluorescent markers. Particles are colored according to the fluorescence intensity of each marker. The maximum fluorescence intensity in each marker was set to 100%.

- It is surprising that the authors explicitly list already the limitations of fluorescence microscopy and super-resolution microscopy in the second paragraph of their introduction, however present a method fully dependent on fluorescence labelling and imaging methods. Actually their approach takes away the spatial information of FM approaches, and further makes the approach prone to the limitations they state.
They are also not fully fair about the limitation they state for Electron microscopy, as newly developed approaches (e.g. doi:10.1093/micmic/ozad067.1091; doi:10.1126/science.aay3134) widely extend the limited field of view and sampling capacity of EM. I recommend the authors to state the potential advantage/superiority of the reported method rather than stating the unclear limitations of the existing powerful methods.

Response:
Regarding fluorescence microscopy, it appears that our description was inadequate and misled the reviewers. There is no problem with fluorescence

microscopy itself. What we intended to convey was that “when attempting to detect individual organelles ‘in cells,’ there are limitations in the resolution of fluorescence microscopy because organelles are densely packed.” To clarify this point, we have added the words “in cells” to the text on Line 49. Also, we thank Reviewer #2 for informing us about high-speed 3D electron microscopy. We have cited the indicated papers in the text, on Lines 54–55, and noted the advance as follows: “except for the recently developed high-throughput electron microscopy.”

- Most organelle markers the isolation of organelles are based on are overexpressed in the cells: endoplasmic reticulum (ER, mTagBFP2 (BFP) – SEC61B), mitochondria (GFP–OMP25 and SNAP–OMP25), and the Golgi (Venus–GS27). This raises significant questions about the native state relevance of the reported results, and how well they represent the endogenous processes.

Response:
To address this concern, we analyzed the behavior of both endogenous and exogenous markers for the same organelles, such as peroxisomes and endolysosomes (S3 Fig and S4 Fig). We found that exogenous PEX3–GFP was distributed in the same cluster as endogenous PMP70, and exogenous GFP–VAMP7 was distributed in the same cluster as endogenous LAMP1. Therefore, we believe that the endogenous process can be monitored using these exogenous (and stably expressed) markers. We have added the following sentences to Lines 132–148.

“Next, we determined whether exogenous markers truly represented endogenous organelles in our multi-parametric particle-based analysis. Organelle particles were prepared from HeLa cells expressing BFP–SEC61B and either GFP–VAMP7 or PEX3–GFP and were labeled with Alexa405–NHS and anti-PMP70–A594 and anti-LAMP1–A680 antibodies (S3 Fig). Particles were extracted from the captured images, and the five- or six-dimensional data were embedded into two-dimensional planes using UMAP. The analysis revealed clusters based on the marker proteins (S4A and S4B Fig) (note that although most of these organelle markers were used in the experiment in Fig 2, independent UMAP generated different distribution patterns). In these UMAP

spaces, exogenous GFP–VAMP7 and endogenous LAMP1 were found in Cluster 3 (S4C Fig). The distribution of GFP–VAMP7 in Cluster 3 was slightly different from that of LAMP1 in Cluster 3. This may reflect preferential localizations of VAMP7 to late endosomes and of LAMP1 to mature lysosomes (REF #28, 29). In addition, exogenous PEX3–GFP co-localized well with endogenous PMP70 in Cluster 2, representing peroxisomes (S4D Fig). These results suggest that even when exogenous organelle markers are overexpressed, UMAP embedding accurately reflects the authentic distribution of these markers.”

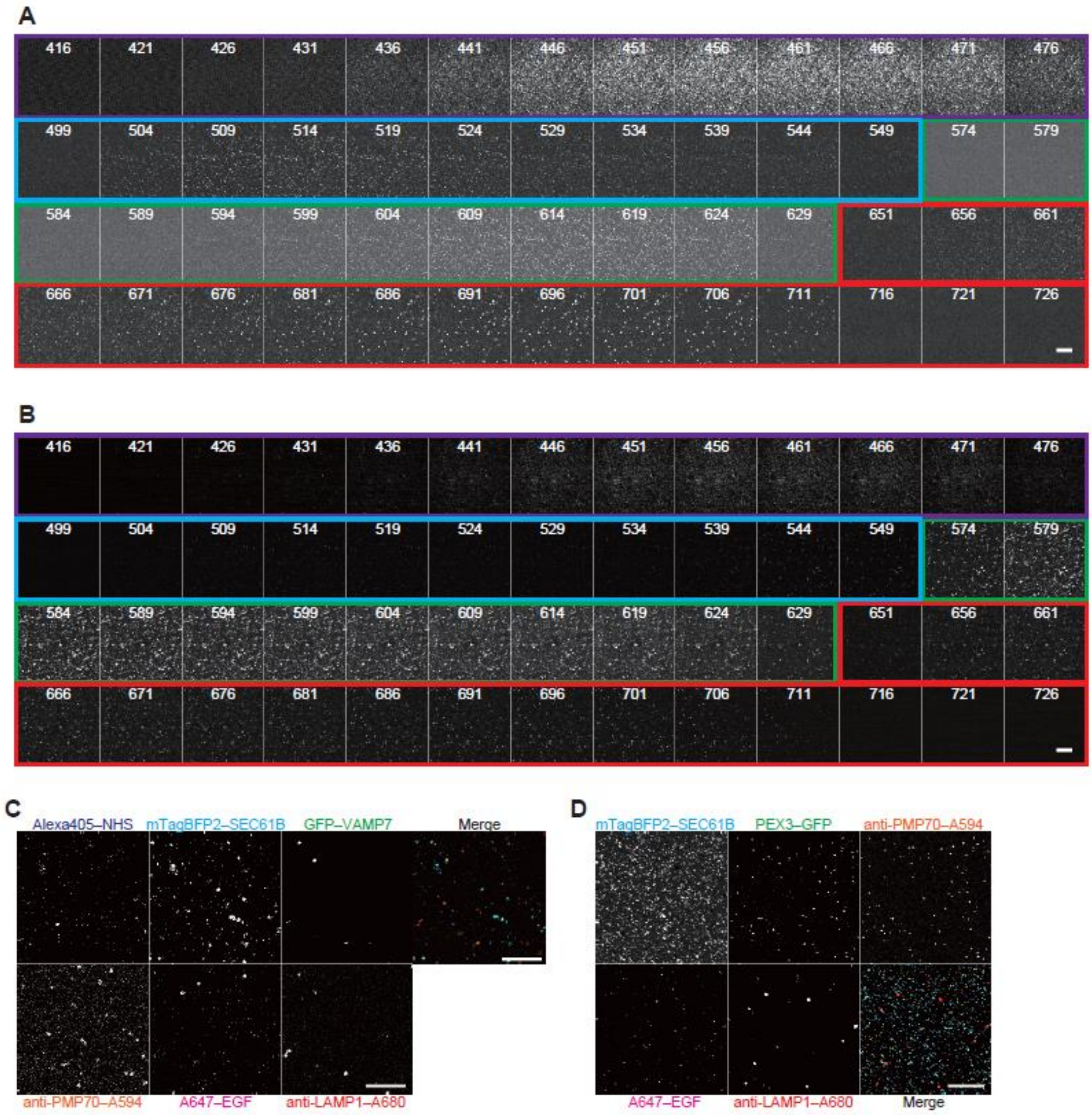

S3 Fig. Spectral imaging and linear unmixing of the images of organelle particles labeled with GFP–VAMP7 or GFP–PEX3.

(A, B) Montage of fluorescence images obtained by spectral imaging of fluorescently labeled organelle particles. Organelle particles from HeLa cells expressing BFP–SEC61B and GFP–VAMP7 (A) or PEX3–GFP (B) were labeled with Alexa405–NHS and both anti-PMP70–A594 and anti-LAMP1–A680 antibodies and are shown as in S1C Fig. Scale bar, 100 μm. (C, D) Unmixing results of the fluorescent spectral images in A and B, respectively. Scale bar, 50 μm.

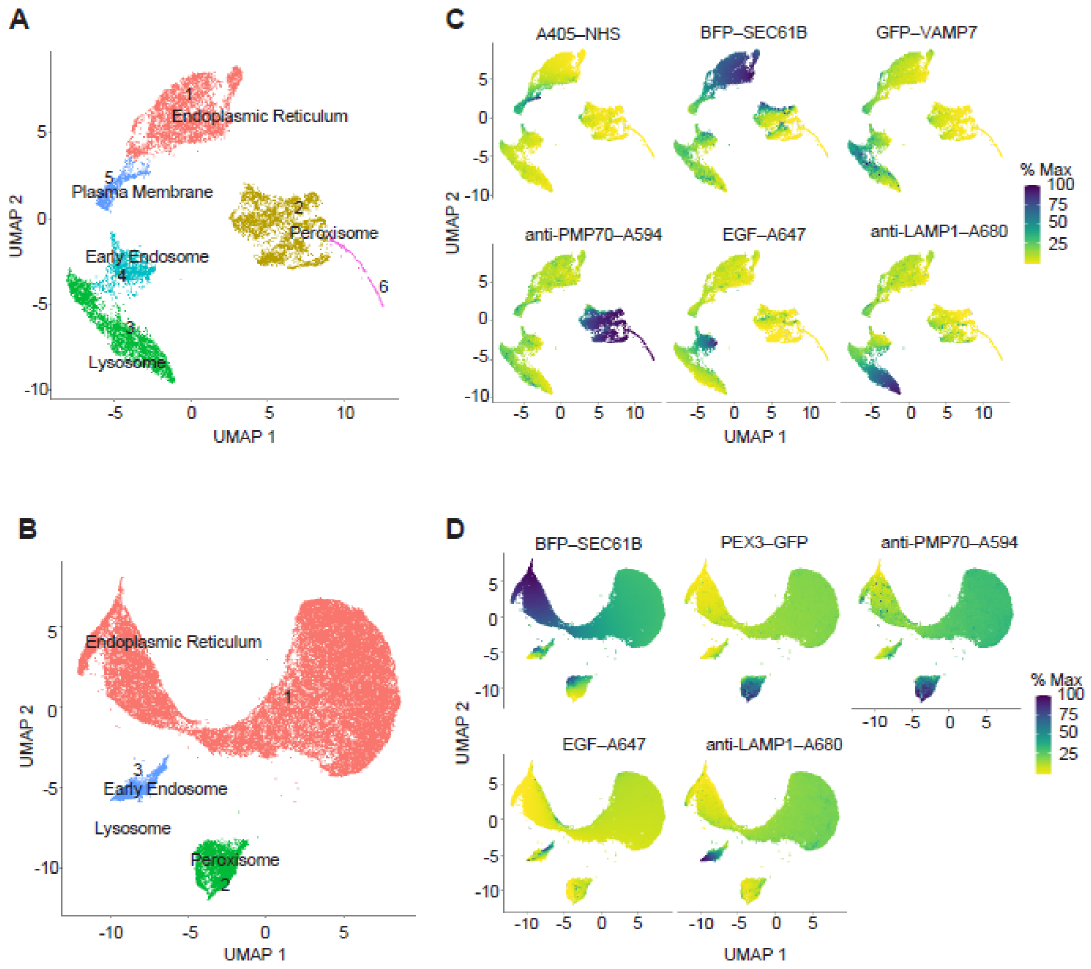

S4 Fig. Multi-parametric single-particle analysis of typical organelles containing GFP–VAMP7 or PEX3–GFP.

(A, B) Uniform manifold approximation and projection (UMAP) embedding of the data obtained from six-color fluorescent images of particles of five typical organelles derived from HeLa cells described in S3 Fig (expressing GFP–VAMP7 (A) or PEX3–GFP (B)). The numbers of particles classified in each cluster in A were as follows: Cluster 1, 3,711; Cluster 2, 3,063; Cluster 3, 2,724; Cluster 4, 1,044; Cluster 5, 786; Cluster 6, 317. The numbers of particles classified in each cluster in B were as follows: Cluster 1, 33,167; Cluster 2, 3,553; Cluster 3, 1,926. (C, D) The intensities of the

fluorescent markers. Particles were colored according to the fluorescence intensity of each marker. The maximum fluorescence intensity in each marker was set to 100%.

- For the application on endosomes, can the authors state what is the new information enabled by their method? They study the very trafficking of EGF and Transferrin, 2 widely used endosomal cargoes with very well characterized trafficking steps, and show they are trafficked through Rab5/7 and Rab11 positive endosomes, respectively. This recapitulates the existing information, however falls short in delivering new insight. The authors can use these cargoes for proof-of-concept, but I would recommend to extend their study with less exploited cargoes to represent the potential of the reported method to deliver new information.

Response:
We thank Reviewer #2 for the positive suggestion about the potential of our method to provide new information. However, demonstrating new biological insights would require considerable time and thus delay the broader application of our methodology, so we would like to submit this manuscript as a Methods paper with the proof-of-concept data. As the Author Guidelines for the *PLOS Biology* article type 'Methods' state that new biological insights are not a requirement for consideration, we believe that our manuscript still satisfies the rigorous publication criteria of the journal.

Reviewer #2 (Significance (Required)):

The significance of biochemical and cellular processes being spatially regulated cellular organelles, and the roles of specific organelles in diseases from cancer to neurodegeneration are continuously being discovered and appreciated. Therefore development of methods reporting on the structure and function of organelles is important to accelerate these studies. In the reported method, however, the ultrastructure (as in Fib 1b) and the spatial information of the cellular organelles are inherently lost. The method falls in between a biochemical and a microscopic approach, however the advantages are not clearly portrayed. I recommend the authors to carefully and explicitly state where their method would be the method of choice rather than a biochemistry,

mass spectroscopy, or microscopy approach. The authors should critically consider such an experiment as a proof-of-concept case.

Response:
We thank Reviewer #2 for the valuable suggestion. We added the following sentences to the Discussion (Lines 311–321).

“A further potential application of our method would be to measure how the levels of key molecules in an organelle change during its differentiation or maturation. For example, the levels of PI4P and syntaxin 17 change during autophagosome maturation (Shinoda et al. eLife Preprint Review doi.org/10.7554/eLife.92189.1), which can be better demonstrated by the present method using multiple markers for each stage of autophagosome formation and maturation, as autophagosomes at different stages coexist in cells. In such cases, our single-particle analysis method, which examines the state of individual autophagosomes, would be more appropriate than biochemical methods that examine averages. In addition, it is difficult to quantitatively analyze many organelle structures in cells using fluorescence microscopy. Our particle-based analysis method can overcome this problem.”

Reviewer #3 (Evidence, reproducibility and clarity (Required)):

Comments, suggestions, and questions

- I would like to start with a positive suggestion. The authors completely miss out on the opportunity to promote their approach by not relying on any type of fixation. In most multiplexing experiments, the first major challenge is to find antibodies that work well for imaging. The second challenge is then to find antibodies that work well under the same fixation conditions. The authors present a multiplexing approach that is completely independent of fixation. I suggest discussing this in the manuscript and promoting the approach in that regard.

Response:

We thank Reviewer #3 for pointing out the advantages of our method. We have added "Our method, which is independent of fixation, is advantageous for the optimization of staining conditions." to the Discussion (Lines 343–344).

- I am wondering what defines the 'resolution' of this approach. I assume it is a combination of the sonication time -the longer the cell is sonicated, the smaller the fragments are - and the density of particles on the coverslip. What are the limits here? How does this affect the UMAP analysis? I would encourage the authors to discuss this in the manuscript.

Response:

The particle density on a coverslip can be easily reduced by simply diluting the particles in a buffer solution. Therefore, there is no density limit, which is an advantage of a cell-free system. To improve the resolution within a single organelle, for example, to separate distinct organellar subdomains, as the Reviewer mentioned, we can prolong the sonication time to make the particles smaller. However, since this would reduce the signal-to-background ratio and destroy organelle contacts, we used sonication conditions that were as mild as possible. To investigate organellar subdomains and fragile contacts, we need to carefully optimize the sonication conditions. We think that this objective will be part of our future work.

We have added the above discussion to the Discussion section, as follows (Lines 332–338).
“Additionally, to improve the resolution within a single organelle, for example, to separate distinct organellar subdomains, we can prolong the sonication time to make the particles smaller. However, because this would reduce the signal-to-background ratio and might destroy organelle contacts, we used sonication conditions that were as mild as possible. To investigate organellar subdomains and fragile contacts, we need to carefully optimize the sonication conditions.”

We do not think that prolonged sonication will affect the UMAP analysis because the relative fluorescent signals of each particle would not change. However, as mentioned above, excessively strong sonication would worsen the signal-to-noise ratio, resulting in poor clustering.

- The only real control the authors present are the correlative light and electron microscopy (CLEM) three images in Figure 1b, which seems very minimalistic for a very central and essential control experiment. How many of these control images did the authors take? Is there possibly a second method for a control experiment to link the fluorescence readout to an organelle fragment (e.g., purification or pulldown)?

Response:
Because all the markers we used are well-established, there should be no concern about the link between the fluorescence readouts and the organelle fragments. We have cited the following papers on Lines 84–85.

SEC61B: Rapoport, T. A., Jungnickel, B. & Kutay, U. Protein transport across the eukaryotic endoplasmic reticulum and bacterial inner membranes. Annu Rev Biochem 65, 271–303 (1996).

OMP25: Horie, C., Suzuki, H., Sakaguchi, M. & Mihara, K. Characterization of signal that directs C-tail-anchored proteins to mammalian mitochondrial outer membrane. Mol Biol Cell 13, 1615–1625 (2002).

GS27: Hay, J. C. et al. Localization, Dynamics, and Protein Interactions Reveal Distinct Roles for ER and Golgi SNAREs. J Cell Biol 141, 1489–1502 (1998).

Fusella, A., Micaroni, M., Di Giandomenico, D., Mironov, A. A. & Beznoussenko, G. V. Segregation of the Qb-SNAREs GS27 and GS28 into Golgi Vesicles Regulates Intra-Golgi Transport. Traffic 14, 568–584 (2013).

Although it is relatively easy to identify mitochondria-derived particles by EM based on their size and the presence of cristae (indeed we see many examples), it is more challenging for other organelles (because they appear as simple vesicles). This is why we showed only mitochondria in Fig. 1b. Furthermore, the main purpose of this EM image is to show membrane contacts between the ER and mitochondria (related to Fig. 3) rather than to show the identity of each organelle.

- Line 37–41: Could the authors please strengthen these statements with an appropriate citation (e.g., a review)?

Response:
We have cited the textbook Molecular Biology of THE CELL (the 6th edition, Chapter 12 and Chapter 13) on Lines 37 and 41.

- Line 51: The statement, “Super-resolution microscopy could partially solve the resolution problem, but it is currently limited to four-color imaging,” is incorrect. Agasti et al. demonstrated up to nine target multiplexed super-resolved imaging with DNA-PAINT in 2017 (https://pubs.rsc.org/en/content/articlehtml/2017/sc/c6sc05420j). Additionally, two papers currently on Biorxiv demonstrate 12 target and 30 target multiplexed super-resolution imaging with FLASH-PAINT (https://www.biorxiv.org/content/10.1101/2023.05.17.541061v1.abstract) and SUM-PAINT(https://www.biorxiv.org/content/10.1101/2023.05.17.541210v1.abstract). Please cite these papers accordingly.

Response:

We thank Reviewer #3 for notifying us of these important studies. We have rewritten the sentence on Lines 51–52 to read “Although multicolor imaging has been attempted with super-resolution microscopy (References #6–#9), it only partially resolves the issue of resolution.”

- The authors use spectral unmixing to overcome the limit of spectral multiplexing. While this has been demonstrated to work well for less than ten targets, it does not scale to multiplexing experiments with more than ten target species. I suggest that the authors discuss in the discussion part of the manuscript the potential of DNA-based multiplexed imaging, such as CODEX or DNA-PAINT, in combination with the presented approach.

Response:
In the Discussion section (Lines 340–344), we have added the sentences, “Current fluorescent particle detection methods utilize spectral multiplexing, but this approach has only been able to detect up to eight colors. Methods with multiplexed imaging, such as CODEX or DNA-PAINT, could significantly increase the number of targets.”

- Regarding the spectral unmixing approach, please cite previous work described in the literature (e.g.,https://www.nature.com/articles/nature22369, or earlier work).

Response:
We thank Reviewer #3 for this information. We have cited the suggested publication on Line 72.

- By using spectral unmixing for multiplexing, this method is limited to confocal due to spectral detection needs and therefore limited in throughput. It would be beneficial if it could work with wide-field type illumination. This could substantially increase the throughput, which is another reason why I think it would be important to discuss sequential multiplexing.

Response:

We agree with the Reviewer's comment. We have added a relevant discussion to Lines 340–344 as described in our response to the above comment from Reviewer #3.

- To image contact sites, the authors use split GFP. There have been discussions that split GFP might, in some cases, facilitate the process that is supposed to be measured, in this case, the formation of contact sites. I suggest using at transient version of split GFP, called split fast, for follow-up experiments in the authors' next papers (https://www.nature.com/articles/s41467-019-10855-0).

Response:
We thank Reviewer #3 for providing this information. We will do as suggested in our next paper.

- Line 27 & 253: Please drop the term 'intuitive' or explain better what you mean by intuitive. For me, UMAP is certainly a very useful tool, but it is not at all what I would describe as intuitive.

Response:
We have deleted 'intuitive' or replaced each instance with a more appropriate alternative in all seven places (Lines 27, 43, 58, 72, 180, 231, and 253 in the previous version of this manuscript).

- Lastly, I want to mention that the authors state they used ChatGPT, DeepL, and DeepL Write for translation from Japanese to English. I appreciate their honesty.

Response:
We thank Reviewer #3 for the comment.

Reviewer #3 (Significance (Required)):

In the manuscript titled "Organelle Landscape Analysis Using a Multi-parametric-Based Method," Kurikawa et al.present a method for multi-

parametric, particle-based analysis of cellular organelles. After lysing cells, the fractions of the organelles are partially labeled with fluorescently tagged antibodies, while others are already tagged with fluorescent proteins, using six to eight spectrally different fluorescent dyes/proteins. These fractions are subsequently immobilized on a poly-L-lysine-coated coverslip. The authors use spectral unmixing to distinguish these markers. The6-8 multiplexed imaging data is then presented in two-dimensional UMAP space. The authors then use this approach to visualize seven major organelles, transitional sites of endocytic organelles, and contact sites between the endoplasmic reticulum and mitochondria using split GFP.
The authors present, in my opinion, a conceptually new and interesting approach by combining spectral unmixing for imaging up to eight targets, with organelle fragment imaging, and presenting multidimensional data in two-dimensional Uniform Manifold Approximation and Projection (UMAP) space in this manuscript. They further validated this approach by linking the results of the experiments to results established or at least reported in the literature.
In general, the manuscript is, in my opinion, a good fit for publication as it presents a conceptionally new approach and an interesting example of applying the UMAP approach, which might be of interest to a broader readership. Therefore, after an appropriate response to my comments, suggestions, and questions (see below), I would recommend this manuscript for publication.

Response:
We thank Reviewer #3 for the positive comment.
